# The effects of bioinformatics preprocessing on cell-free DNA fragment analysis

**DOI:** 10.1093/gigascience/giaf139

**Published:** 2025-10-30

**Authors:** Ivna Ivanković, Zsolt Balázs, Todor Gitchev, Cécile Trottet, Norbert Moldovan, Idris Bahce, Florent Mouliere, Michael Krauthammer

**Affiliations:** Department of Quantitative Biomedicine, University of Zurich, Zurich 8057, Switzerland; University Hospital Zurich, Zurich 8091, Switzerland; Department of Quantitative Biomedicine, University of Zurich, Zurich 8057, Switzerland; University Hospital Zurich, Zurich 8091, Switzerland; Department of Quantitative Biomedicine, University of Zurich, Zurich 8057, Switzerland; University Hospital Zurich, Zurich 8091, Switzerland; Department of Quantitative Biomedicine, University of Zurich, Zurich 8057, Switzerland; University Hospital Zurich, Zurich 8091, Switzerland; Department of Pathology, Amsterdam UMC, Vrije Universiteit Amsterdam, Cancer Centre Amsterdam, Amsterdam 1007 MB, The Netherlands; Imaging and Biomarkers, Cancer Center Amsterdam, Amsterdam 1081 HV, The Netherlands; Department of Pulmonology, Amsterdam UMC, Vrije Universiteit Amsterdam, Cancer Centre Amsterdam, Amsterdam 1081 HV, The Netherlands; Department of Pathology, Amsterdam UMC, Vrije Universiteit Amsterdam, Cancer Centre Amsterdam, Amsterdam 1007 MB, The Netherlands; Imaging and Biomarkers, Cancer Center Amsterdam, Amsterdam 1081 HV, The Netherlands; Cancer Research UK National Biomarker Centre, University of Manchester, Manchester M20 4BX, United Kingdom; Department of Quantitative Biomedicine, University of Zurich, Zurich 8057, Switzerland; University Hospital Zurich, Zurich 8091, Switzerland

**Keywords:** cell-free DNA, fragmentomics, bioinformatics workflow, benchmarking

## Abstract

**Background:**

While cell-free DNA (cfDNA) is a promising biomarker for cancer diagnosis and monitoring, there is limited agreement on optimal cfDNA collection and extraction protocols as well as analysis pipelines of the corresponding cfDNA sequencing data. In this article, we address the latter by studying the effect of various bioinformatics preprocessing choices on derived genetic and epigenetic cfDNA features and study how observed feature differences influence the downstream task of separating between healthy and cancer cfDNA samples.

**Results:**

Using low-pass whole-genome cfDNA sequencing data from 20 lung cancer and 20 healthy samples, we assessed the influence of various preprocessing settings, such as read trimming, filtering of secondary alignments, and choice of genome build, as well as practices such as downsampling or selecting for a short fragment on derived cfDNA features, including cfDNA fragment size, fragment end motifs, copy number alterations, and nucleosome footprints. Our results demonstrate that the analyzed features are robust to common preprocessing choices but exhibit variable sensitivity to sequencing coverage. Fragment length statistics and end motifs are the least affected by low coverages, whereas nucleosome footprint analysis is very sensitive to them. Our findings confirm that selecting for shorter fragments enhances cancer-specific signals but, by removing data, also reduces signals in general. Interestingly, we find that fragment end motif analysis benefits the most from *in silico* size selection. We also observe that the filtering of low-quality and secondary alignments and choice of genome build result in slight improvements in cancer classification performance based on nucleosome coverage and copy number features.

**Conclusions:**

Altogether, we conclude that cfDNA analysis is minimally affected by different bioinformatics preprocessing settings, but we describe some synergistic effects between analytical approaches, which can be leveraged to improve cancer detection.

## Introduction

Cell-free DNA (cfDNA) fragments are released into the bloodstream during cell death and carry genetic and epigenetic information from their cells of origin [1–3]. The analysis of cfDNA emerged as a valuable diagnostic tool in cancer detection and screening, since sample collection is minimally invasive and cfDNA fragmentation patterns provide valuable clinical insights [4]. In healthy individuals, cfDNA is mainly derived from hematopoietic cells, while in patients with cancer, a fraction of this cfDNA is released from the tumor cells, known as circulating tumor DNA (ctDNA) [5, 6].

The use of next-generation sequencing technologies has advanced the investigation of fragmentomic and genomic profiles of cfDNA-based liquid biopsies [7]. A substantial portion of cfDNA research is directed toward identifying and studying characteristics of cfDNA through low-coverage sequencing data, offering a cost-effective approach for cancer detection, monitoring disease progression, and treatment response [8]. cfDNA fragments derived from tumors are generally shorter than those originating from hematopoietic cells [9, 10]. Estimating copy number aberrations from low-coverage sequencing data provides a cost-effective solution for calculating the tumor fraction (portion of ctDNA in the total cfDNA). Furthermore, cfDNA fragmentation is nonrandom and can differ depending on the epigenetic environment and gene expression in the cell types of origin [11, 12]. Nonrandom fragmentation affects not only the genomic location of the cfDNA fragments but also the sequences at the end of fragments [13–15]. These distinctive features, including fragment length, fragment end motif, nucleosome positioning, and coverage at specific genomic regions, not only facilitate the differentiation between diseased and healthy individuals but also offer information about the cell type of origin and thus have a great potential to serve as cfDNA biomarkers.

While the clinical benefits of studying cfDNA have been established, a systematic comparison of both experimental and computational methods is essential to develop and standardize best practices in cfDNA analysis. Efforts toward standardizing cfDNA measurements have been made by comparing the DNA extraction kits [16] and studying the effects of preanalytical and physiological variables on cfDNA fragmentation [17]. Even though many software tools have been developed for the analysis of cfDNA genetic and fragmentomic features (a list of such tools is collected on the NucPosDB website [18]), the effects of various bioinformatics preprocessing approaches on these features have not been thoroughly examined to this date.

Prior works on tissue biopsy highlighted the effects of bioinformatics preprocessing on the analysis of sequencing data, such as variant calling, gene expression analysis, and copy number variation analysis [19–21]. Accurate alignment in variant calling analyses hinges on the choice of the reference genome, read trimming, and postalignment filtering, as mismatches can lead to false variant calls [22, 23]. A recent study [24] found different genome aligners and trimming options on cfDNA fragment length and end motifs. These findings further highlight that the careful optimization of these preprocessing steps is imperative to ensure robust and meaningful biological insights from cfDNA genome-wide sequencing.

Here, we systematically evaluate the effects of bioinformatics preprocessing on the analysis of whole-genome cfDNA sequencing data. We present cfDNA-Flow, a modular bioinformatics pipeline offering a range of preprocessing options. Using cfDNA-Flow, we comprehensively evaluate the effects of preprocessing settings on cfDNA sequencing data. We evaluate our results according to how each modality differs between healthy and cancer cfDNA samples and whether they impact the performance of liquid biopsy assays.

## Data Description

### Patient recruitment and sample processing

Patient and healthy individual samples were recruited following informed consent via the Liquid Biopsy Center at the Amsterdam UMC, at locations VUmc and AMC (study approved by the Amsterdam UMC ethics board, METC U2019_035). Blood samples were processed as previously described [25]. In brief, blood was collected in EDTA tubes and processed via a double-centrifugation protocol (900 × *g* for 15 minutes, 2,500 × *g* for 10 minutes), and supernatant plasma was stored at–80°C. DNA was isolated using the QIAsymphony DSP Circulating Nucleic Acids kit (QIAgen). cfDNA was quantified using a cfDNA kit and a Tapestation 4200 system (Agilent). Indexed sequencing libraries were prepared using 1 to 10 ng DNA and the ThruPLEX Plasma-seq kit (Takara). Libraries were pooled in equimolar amounts and sequenced to >1× coverage on a NovaSeq 6000 (Illumina), generating 150-bp paired-end reads from a S4 flowcell.

## Analyses

### Overview

**Figure 1:**
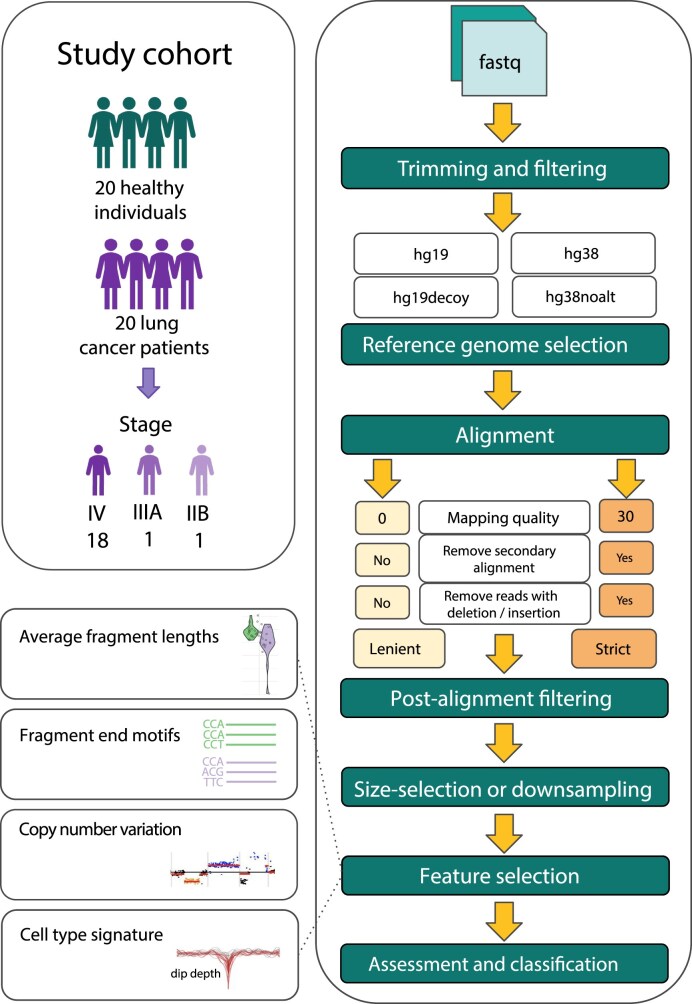
Overview of study cohort and cfDNA preprocessing workflow. We analyzed the low-pass whole-genome cfDNA sequencing data of 20 healthy individuals and 20 patients with lung cancer. Following read trimming and filtering, alignment to the chosen reference genome, and postalignment filtering using lenient or strict options, we extracted 4 types of features to enable the classification of healthy and cancer samples: fragment lengths, tumor fraction scores derived from copy number aberrations, and cell-type signatures.

We built a pipeline for reproducible analysis using a scalable bioinformatics workflow engine, Snakemake [26], which enabled us to preprocess samples testing different settings (Fig. 1) and different postalignment filtering options and extract biologically relevant features. We applied our pipeline to a cohort of 20 healthy and 20 late-stage lung cancer samples (18/20 stage IV) (Fig. 1). Their tumor fraction, estimated based on the amplitude of copy number aberrations using ichorCNA, ranged between 0.6% and 84%, with a mean of 15% and a detection threshold of 3%. Raw read coverage ranged between 0.04× and 10.9×, with a mean of 2.5×. From all samples, we extracted 6 features covering 4 distinct modalities, which have been used to classify tumor and healthy plasma cfDNA samples: (i) cfDNA fragment length, (ii) ichorCNA tumor fraction, and (iii) tMAD score based on copy number alterations, as well as (iv) fragment end motifs and nucleosome coverage over (v) blood-cell specific and (vi) cancer-specific DNase hypersensitivity sites (DHSs) [4, 10, 27, 28].

### The effects of bioinformatics preprocessing on fragment count statistics

We investigated reads removed during the following bioinformatics preprocessing steps: quality trimming, alignment, and postalignment filtering. We found that trimming and discarding reads shorter than 50 bp after trimming reduced the number of reads by 2%. The read count loss between trimmed and nontrimmed files was less prominent after alignment and postalignment filtering, because many reads that would have needed extensive trimming were either not mapped or mapped ambiguously. The choice of genome build did not affect the read count substantially. We tested 2 postalignment filtering options: lenient filtering, where we removed only unmapped and duplicate reads, and strict filtering settings, where, in addition, we removed low-quality, indel-containing reads and any read that had a secondary alignment. On average, discarded fragments were shorter than kept fragments (Supplementary Fig. S1A). It has been observed that cancer-derived cfDNA fragments tended to be short [10], but the discarded reads were not enriched in tumor-derived cfDNA, as copy number analysis–based tumor fraction estimates were not increased when analyzing the discarded reads (Supplementary Fig. S1B). Moreover, the reads discarded by strict filtering even showed a decreased tumor fraction estimate for the cancer samples and an increased estimate for the healthy samples, which confirms that these discarded reads are not helpful in distinguishing cancer and healthy samples.

### The effects of bioinformatics preprocessing on genetic and epigenetic cfDNA features

**Figure 2:**
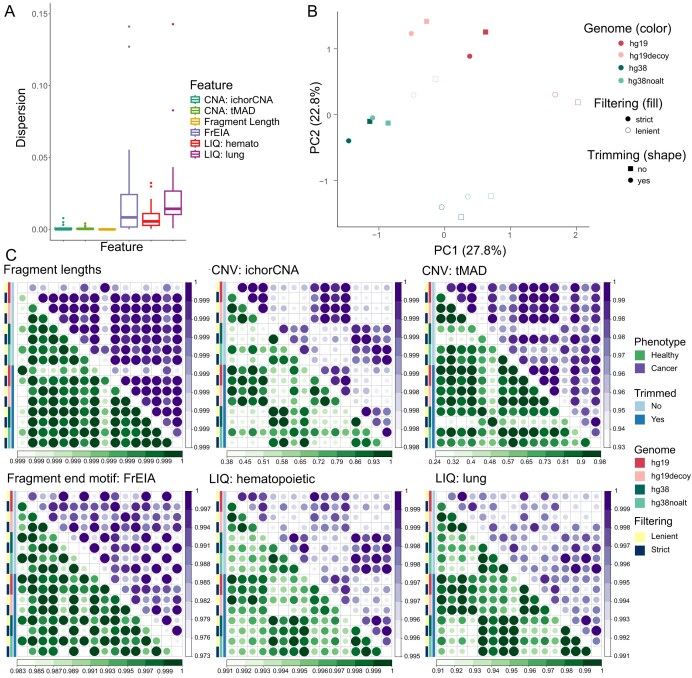
Variance and characteristics of studied features across different preprocessing settings. (A) Dispersion (variance divided by the mean) of examined features among 16 studied preprocessing settings. Fragment length feature displays the lowest dispersion, whereas lung epithelial cell-type signature displays the highest sample dispersion across examined preprocessing settings. (B) A principal component analysis (PCA) plot of 6 investigated features across 16 studied preprocessing settings. Filtering and the choice of reference genome build appear to explain the most variance in the data. Trimming has the least effect. (C) Correlation plots for 6 examined features, displaying Pearson’s correlation of the feature’s value obtained by 1 preprocessing setting with values obtained by every other preprocessing setting. The lower diagonal represents healthy samples (green), while the upper diagonal corresponds to lung cancer samples (purple). The color legends represent 4 distinct categories of investigated bioinformatics preprocessing settings: trimming, choice of reference genome, and postalignment filtering.

All 6 of the studied features appeared robust to the 16 examined preprocessing settings, with no statistical difference in their mean values observed (analysis of variance, *P* = 1). The studied features exhibited low variance, with lung epithelial cell-type signature, fragment end motifs, and copy number aberration (CNA)–derived features displaying slightly higher variance than fragment lengths and hematopoietic cell-type signature (Fig. 2A). Most of the observed variance was explained by filtering and choice of the reference genome, while trimming had the least effect (Fig. 2B).

Even though we did not observe any significant difference in the means of features derived by 16 studied preprocessing settings, noticeable patterns emerged after correlating the values of a feature calculated using 1 preprocessing setting with those calculated using every other preprocessing setting (Fig. 2C). Features derived from CNAs, such as tumor fraction and nucleosome footprints, indicated possible dependence on the choice of reference genome, and fragment end motifs’ FrEIA scores showed a higher correlation if the same alignment filtering was applied.

### The effects of bioinformatics preprocessing on classifying cancer and healthy samples

**Figure 3:**
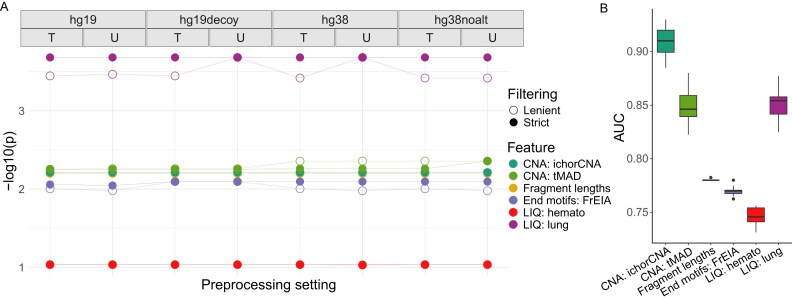
Extracted cfDNA features in healthy and cancer samples and their differences in distinguishing healthy from cancer samples across 16 bioinformatics preprocessing settings. (A) The −log_10_(*P*) values of examined features evaluated across 16 preprocessing settings. Higher values indicate better separation between healthy individuals and patients with lung cancer. The *P* values were computed using a *t*-test. (B) The area under the ROC curve (AUC) for 6 investigated features across 16 different bioinformatics preprocessing settings. A higher AUC value indicates better distinction between the healthy and cancer samples. Boxes represent interquartile range (25th and 75th percentiles), with the median depicted as a black line. The whiskers extend to display the minimum and maximum values

We performed *t*-tests to compare each feature’s ability to differentiate cancer and healthy samples across 16 studied preprocessing settings (Fig. 3A). Interestingly, the greatest difference between the means of cancer and healthy samples was observed in lung epithelial cell-type signatures and the least in the hematopoietic cell-type signatures, across all settings. Copy number analyses using ichorCNA and tMAD achieved similarly good separation to using fragment length averages and fragment end motifs. Then, we classified samples based on each feature individually (Fig. 3B) to further investigate the effects of preprocessing settings on studied features and the distinction between the healthy and cancer samples. Tumor fraction calculated using ichorCNA showed the highest, whereas hematopoietic cell signature showed the lowest area under the curve (AUC) scores. The effects of the investigated preprocessing decisions (trimming, reference genome choice, alignment filtering) on distinguishing healthy and cancer samples are detailed in the paragraphs below.

**Trimming:** Read trimming had no impact on the observed average fragment length features derived from copy number aberrations and, surprisingly, not even on fragment end motifs (Fig. 3A). Nucleosome footprint analysis was slightly but not significantly affected by trimming the reads (lung *P*-adjusted = 0.1056 and hematopoietic cell signatures *P*-adjusted = 0.1554) (Supplementary Fig. S2).
**Reference genome choice:** The choice of reference genome had no impact on the fragment lengths or cell-type–specific nucleosome signatures (Fig. 3A). CNA-derived features, especially CNA calculated by ichorCNA, were significantly affected by the choice of reference genome, showing better performance in distinguishing between healthy and cancer samples in the hg38 and hg38noalt genomes compared to the hg19 and hg19decoy genomes (Fig. 3A, Supplementary Fig. S3). Lung epithelial signatures differed slightly (not significantly) more between healthy and lung cancer samples when using the hg19 or hg19decoy genome builds. However, it has to be noted that to determine the cell-type–specific nucleosome footprints, we used a list of DHSs that was annotated on the hg19 genome, and coordinates were converted to the hg38 build using Liftover (see Methods).
**Alignment filtering:** Strict filtering increased the differences between healthy individuals and patients with cancer when measuring fragment lengths (*P*-adjusted = 0.00004) and fragment end motifs (*P*-adjusted = 0.0162; Fig. 3A, Supplementary Fig. S5). Furthermore, nucleosome footprint analysis achieved slightly, albeit not significantly (*P*-adjusted = 0.1104 for lung epithelial and *P*-adjusted = 0.1134 for hematopoietic signatures), better distinction of the healthy and cancer cohort when applying strict alignment filtering.

### The effects of downsampling and size selection on classifying cancer and healthy samples

Best practices often call for downsampling the input samples to the same coverage to normalize for the signal-to-noise ratio, which might bias downstream inference. To test the effects of downsampling and also the coverage dependence of the tested features, we downsampled our dataset to uniformly 0.1× and 1× coverage and compared the results to those of the nonuniform full-coverage dataset. As short fragments have been shown to be more likely to originate from cancer cells [10], we also performed *in silico* selection of short fragments (<150 bp) and analyzed the same features.

**Figure 4:**
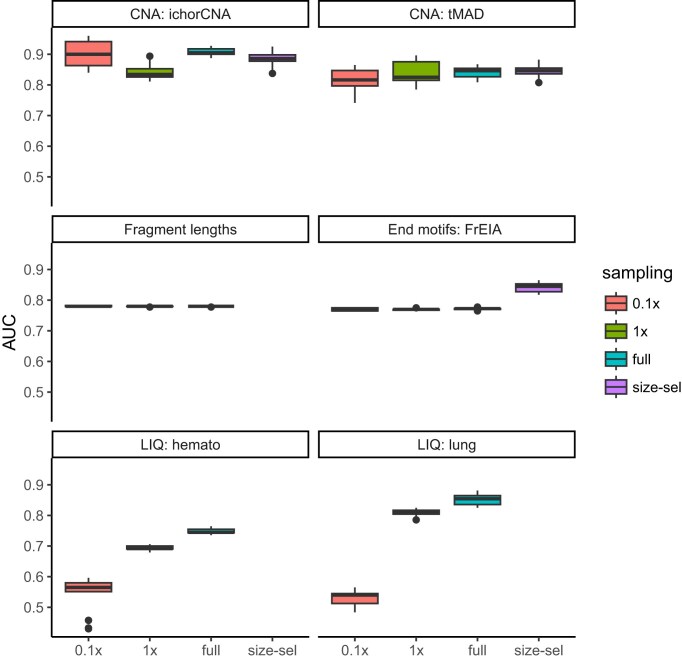
Coverage dependence of the investigated features and the effects of size selection. The area under the ROC curve (AUC) for 6 investigated features across 16 different bioinformatics preprocessing settings. A higher AUC value indicates better distinction between the healthy and cancer samples. 0.1×, 1×, and full refer to the coverage of the samples. Size-sel: *in silico* size selection of fragments <150 bp. Boxes represent interquartile range (25th and 75th percentiles) with the median depicted as a black line. The whiskers extend to display the minimum and maximum values.

**Downsampling:** While features such as fragment length averages and fragment end motifs were robust to downsampling, copy number analysis using both ichorCNA and tMAD became noisier at lower coverages (Supplementary Fig. S5B), although cancer-healthy classification remained accurate even at 0.1× coverage, consistent with earlier publications [27] (Fig. 4). Nucleosome footprints analyzed by LIQUORICE were very sensitive to coverage (Supplementary Fig. S6), with AUC values only slightly better than random chance (Fig. 4).
**Size selection:** As selecting for short fragments (<150 bp) in our dataset results in very low-coverage data (<0.1×), we could not evaluate the effects of size selection on nucleosome footprints. Investigating the effects of size selection on copy number analysis, we found that while the average tumor fraction estimates (Fig. 4) increased upon size selection, the accuracy of cancer-healthy classification, on the other hand, did not. This is because in some cancer samples, size selection did not increase the tumor fraction estimates, but the very low coverage of the data increased noise. Most interestingly still, we found that cancer-healthy classification based on fragment end motifs was substantially improved by size selection. We attribute this result to the selective enrichment of cancer-specific fragment end motifs of shorter reads. The detection of these motifs was not hindered by the very low coverage of the dataset as fragment end motif analysis is generally robust to coverage.

### Computational resources and runtimes

Computation runtimes for the preprocessing steps ranged between 9.5 and 14 hours (median = 11.4 hours) for 40 analyzed samples (Supplementary Fig. S7). Peak RAM usage was observed during the alignment step where it exceeded 16 to 18 GB per core. Trimmed and untrimmed reads showed no significant CPU usage difference likely due to high-quality raw reads.

## Discussion

We built a versatile bioinformatic pipeline, cfDNA-Flow, a reproducible workflow to analyze different cfDNA features, offering flexibility in the choice of preprocessing settings. cfDNA-flow sets itself apart from earlier pipelines such as cfDNApipe [30] and cfDNAPro [24] by being implemented in Snakemake, a reproducible workflow management system. The recent cfDNA UniFlow [31] is also implemented in Snakemake, but it focuses more on extracting GC-bias–corrected coverage values from genomic regions (while also running ichorCNA). In contrast, cfDNA-Flow enables the extraction of a wider range of cfDNA-related features using third-party applications. Overall, cfDNA-Flow is designed to facilitate benchmarking studies using whole-genome cfDNA sequencing data. Using cfDNA-Flow, we benchmarked commonly used bioinformatics preprocessing settings on a low-pass whole-genome sequencing dataset containing plasma cfDNA of 20 healthy individuals and 20 patients with lung cancer. We derived genetic, fragmentomic, and epigenetic features from the cfDNA data and assessed the effects of the preprocessing settings on those features. Our principal findings underscore the robustness of studied genetic, epigenetic, and fragmentomic features across the 16 preprocessing settings investigated. We detected no statistically significant differences in the mean values of these features across different preprocessing settings.

Although we found that most of the examined preprocessing settings did not substantially change the outcome of the analyses, we found a slight advantage to using recent genome builds (hg38 vs. hg19) when performing copy number analysis. We believe that the better curated genome build allowed for fewer misaligned reads, which ultimately led to a better performance of these tools with regard to classifying cancer. However, as not every annotation is available for all genome builds, users may still be limited to the use of genome builds that have the annotation they are interested in. We found that nucleosome footprint analysis, which relied on preexisting annotation, distinguished healthy and cancer samples better when the annotation matched the genome build of the annotation. This is possibly caused by inaccurate conversion between genome build coordinates [32]. Interestingly, we did not observe a difference in aligning to genomes with decoy or alternative contigs and more minimalist genome builds of the same version (e.g., hg38 vs hg38noalt). Further, we observed that strict alignment filtering (i.e., removing secondary alignments and reads with poor mapping quality or that align with indels) improved the distinction of patients with lung cancer and healthy individuals when analyzing fragment lengths, fragment end motifs, and, to a lesser degree, nucleosome footprints.

By performing downsampling, we confirmed previous publications’ conclusions that (i) fragment length– and fragment end motif–based analyses are not affected by sequencing coverage; (ii) accurate copy number analysis calling requires >1× coverage, but cancer-healthy classification is feasible with 0.1× coverage data at least with late-stage cancer cohorts; and (iii) nucleosome footprinting requires high (>1×) coverage input. Investigating the effects of size selection, we found that enriching for cancer-specific fragments and greatly reducing the coverage creates a trade-off for copy number analysis that has to be taken into consideration. Importantly, however, fragment end motif analysis is not sensitive to coverage but benefits from the enrichment of cancer-specific fragments and therefore can only benefit from selecting for shorter fragments *in silico*. To provide practical guidance for future studies, we summarize our recommendations for preprocessing choices in Table 1. Besides emphasizing the coverage dependency of certain cfDNA features, we are also highlighting the importance of using the most complete reference genomes for CNA. Furthermore, we recommend strict alignment filtering (removal of secondary alignments and low-quality mappings) and the use of a matching reference genome and an annotation for regions of interest.

**Table 1:** Recommended preprocessing options for cfDNA fragmentomic analyses concluded from our analyses. Coverage refers to the lowest fragment coverage at which the analyzed features yielded stable results in our downsampling benchmarks. Read trimming did not substantially influence the analysis of the measured features, and therefore, it is not shown here.

Feature	Coverage	Preprocessing	Reference genome	Notes
Fragment length statistics	0.1×	Strict filtering	—	Robust to most preprocessing settings
Fragment end motifs (FrEIA)	0.1×	Strict filtering	—	Size selection (<150 bp) beneficial
Copy number alterations (ichorCNA, tMAD)	1×	—	hg38 > hg19	Size selection (<150 bp) highlights tumor-specific CNAs, but low coverage introduces noise
Nucleosome footprints (LIQUORICE)	>1×	—	Use reference matching the annotation	Very sensitive to coverage

Limitations of our study include that we have analyzed a relatively small dataset and that the cancer samples were of patients with late-stage lung cancer. We focused our analyses on late-stage cancer samples, with clear cancer-specific alterations, to provide sufficient signals for analysis of various features, as early-stage cancers are often undetectable from liquid biopsy data [33, 34]. Furthermore, the focus of our analysis was low-coverage whole-genome sequencing, which can output copy number variants and fragmentation data. Our analysis, therefore, did not include methylation-related or mutation-related data, which are also among the most intensively investigated areas of liquid biopsy [35–37]. However, we emphasize that there is a lack of computational benchmarking on mutation and methylation analyses, and those are likely affected differently by preprocessing than the features analyzed in our study. Further research in the field of DNA methylation and mutations could expand and complement our findings on copy number variants and fragmentation data.

In conclusion, we developed a modular reproducible cfDNA-sequencing preprocessing workflow called cfDNA-Flow and evaluated several bioinformatics preprocessing settings for the analysis of low-pass whole-genome cfDNA sequencing data. Our findings show that most preprocessing settings have little impact on downstream analysis, with only strict alignment filtering, improving the detection of cancer samples. In general, our recommendations are to use strict alignment filtering to remove any ambiguous alignments and to use genome builds with the appropriate annotation for any downstream tasks.

## Methods

### Preprocessing

For computing, we used 16 CPUs and 18 and 16 GB RAM per core for the alignment and the downstream analysis, respectively.

#### Read trimming

To test the effects of trimming on the analysis of cfDNA sequencing data, trimming was performed using the skewer [38] tool (v0.2.2), an efficient trimmer for paired-end reads. We explored 2 settings: (i) no read quality filtering or trimming and (ii) read quality filtering and trimming with the following values: lowest mean quality of the read (*Q*) allowed before trimming was set to 30, minimum length allowed after trimming (*l*) to 50 bp, and trimming the 3′ end of the reads until the quality (*q*) of at least 35 was reached.

#### Reference genome choice and mapping

We carried out our analyses on sequencing reads aligned to 4 different human reference genome builds—hg19, hs37d5, hg38, and hg38— without alternative contigs (for download links, see the Data availability section). Mapping against the reference genome was performed using the Burrows–Wheeler aligner (BWA) software package’s [39] (v0.7.17-r1188) bwa-mem algorithm. Duplicates are marked using Picard tools (v2.27.1) from the Genome Analysis Toolkit [40] (v4.2.6.1).

#### Quality control

The quality of raw FASTQ files was assessed using FastQC (v0.11.9), and alignment summary metrics were calculated using the Picard toolkit. A MultiQC [41] (v1.13.dev0) report was generated for both FastQC and Picard alignment metrics.

#### Postalignment filtering

To test the effects of postalignment filtering, we explored the following postalignment filtering options: strict filtering (removing unmapped reads, reads that are or have a secondary alignment, reads with mapping quality <30, and reads with deletions or insertions in them) and lenient filtering (removing only unmapped reads).

#### Analyzing the effects of read filtering

To analyze properties of reads filtered out during trimming and alignment filtering, we studied and compared discarded and kept reads. We used the “diff” option from the bamUtil (v1.0.15) [42] repository to create BAM files containing only discarded reads by finding a difference between the unfiltered BAM file and the final BAM files after preprocessing. We downsampled preprocessed BAM files containing kept reads to the coverage of newly generated BAM files containing the reads filtered out during the analysis. Subsequently, we calculated tumor fraction using ichorCNA [27] for kept reads and discarded reads both selecting for short fragments (20–150 bp) and keeping fragments of all sizes. Additionally, we calculated and compared the fragment lengths in BAM files with discarded and kept reads.

### Feature extraction

#### Fragment length features

We calculated mean, median, and standard deviation values for fragments sized 100 to 220 bp, which correspond to the mononucleosomal size range. The size range corresponds to fragments originating predominantly from mononucleosomes. We also calculated the frequencies of cfDNA fragment sizes ranging from 70 to 1,000 bp in 10-bp bins.

#### Copy number analysis

We used copy number analysis tools, ichorCNA (v0.2.0) [27] and tMAD [10], to estimate copy number changes and tumor fraction. We created a panel of normals using the 20 healthy samples and ran ichorCNA assuming diploid state and copy numbers up to 3 with *a priori* estimates of normal fraction set to 0.5, 0.9, 0.95, 0.99, 0.995, and 0.999.

#### Fragment end motifs

We used the FrEIA tool [15] with default settings. However, preprocessing steps (such as trimming) otherwise performed by FrEIA were skipped to allow for the comparison of results between the different preprocessing approaches. Cancer and control fragment end motifs were determined once, using the original dataset.

#### Differential coverage analysis over DNase hypersensitivity sites

We examined differential coverage over DHSs in hematopoietic and small airway epithelial cells using the LIQUORICE software (v0.5.4) [43]. Region sets specific for cell types were defined using cell-type–specific clusters of DHSs based on data from the University of Washington [44] and Duke ENCODE groups [45]. The downloaded regions were coordinates on the hg19 genome, which were converted to hg38 coordinates using Liftover [46]. LIQUORICE queries the average coverage and models the dip in coverage over the specified region sets, presuming the data can be best described as a sum of 3 Gaussian distributions. The calculated dip depth values are compared to a control group (healthy cohort) and *z*-scaled. We used the *z*-scaled coverage dip depth values as cell-type–specific signatures.

#### Downsampling and size selection

Both downsampling and size selection were performed using samtools view [47, 48]. For downsampling, the coverage of a given sample was calculated, and samtools view’s subsampling proportion was calculated to achieve 0.1× and 1× coverages. Size selection was performed only retaining fragments shorter than 150 bp.

#### Reproducibility and scalability

We developed the cfDNA-Flow pipeline using Snakemake [26] software for scalable and reproducible analysis, following best code development guidelines. Snakemake enables the user to use the pipeline on single-core workstations or compute clusters without modifying the code. We also provide a Singularity [49] recipe for building a container that has all needed packages and tools to run all pipeline steps. The architecture also allows cloning and running the pipeline code using tools from the Singularity container.

## Ethics, Consent, and Permissions

We report on data collected for the study METC U2019_035, which was approved by the Amsterdam UMC ethics board. Informed consent was obtained from all study participants.

## Availability of Source Code and Requirements

Project name: cfDNA-Flow

Project homepage: https://github.com/uzh-dqbm-cmi/cfDNA-Flow (source code for figures in this publication can be found at https://github.com/uzh-dqbm-cmi/cfDNA-Flow_paper)

License: GPL-v3 (and CC0 for the source code of the figures)

Operating system: Linux

Programming language: Python, R, Shell

Package management: Conda

Hardware requirements: At least 128 GB memory (for alignment) and 8 CPU cores recommended

WorkflowHub: https://doi.org/10.48546/workflowhub.workflow.1900.1

## Additional Files

**Supplementary Fig. S1**. (A) The average fragment lengths of discarded reads are shorter than those of kept reads. (B) Tumor fractions calculated with ichorCNA were compared across different preprocessing settings for both kept and discarded reads, considering all reads as well as only short reads. It was observed that discarded reads do not show enrichment in tumor DNA, as their tumor fractions do not increase compared to those of cancer samples with kept reads. *Downsampled to match the coverage of BAM files containing only short reads.

**Supplementary Fig. S2**. The effect of trimming on distinguishing healthy and cancer samples. The *t*-statistics from *t*-tests comparing healthy and cancer samples were calculated for various features, including tumor fraction estimated by ichorCNA, tMAD score, average fragment length, hematopoietic and cell-type signatures from LIQUORICE, and normalized coverage. Each data point represents a unique preprocessing setting combination (16 per feature).

**Supplementary Fig. S3**. The e ect of genome build on distinguishing healthy and cancer samples. The *t*-statistics from *t*-tests comparing healthy and cancer samples were calculated for multiple features, including tumor fraction estimated by ichorCNA, tMAD score, average fragment length, hematopoietic and cell-type signatures from LIQUORICE, and normalized coverage. Each data point represents a unique preprocessing setting combination (16 per feature). Significance values correspond to results of pairwise *t*-tests, corrected for multiple hypothesis testing using the Bonferroni method.

**Supplementary Fig. S4**. The e ect of GC-bias correction on distinguishing healthy and cancer samples. The *t*-statistics from *t*-tests comparing healthy and cancer samples were calculated for several features, including tumor fraction estimated by ichorCNA, tMAD score, average fragment length, hematopoietic and cell-type signatures from LIQUORICE, and normalized coverage. Each data point represents a unique preprocessing setting combination (16 per feature). Significance values correspond to results of pairwise *t*-tests, corrected for multiple hypothesis testing using the Bonferroni method.

**Supplementary Fig. S5**. The e ects of downsampling and size selection on copy number analysis. (A) Tumor fraction estimates output by ichorCNA for healthy (green) and cancer samples (purple). The boxes depict interquartile ranges. For each setting, *n*(healthy) = 20 and *n*(cancer) = 20. (B) Selected copy number plots from 2 patients with cancer, generated with ichorCNA. LP0028_01_L004’s copy number analysis was improved by selecting for short fragments (<150 bp), whereas LP0026_42_L004’s was not.

**Supplementary Fig. S6**. The e ects of downsampling on nucleosome footprint analysis with LIQUORICE. Four patients’ LIQUORICE plots are shown. The left columns show coverage values at hematopoietic-specific promoter regions and the right columns at lung epithelial-specific promoter regions. Green: healthy control. Blue: cancer sample’s dip in coverage significantly less than in controls. Red: cancer sample’s dip in coverage significantly more than in controls. Gray: cancer sample not significantly different from controls. The plots show that cell-type signatures are clear in the full-coverage data but are very noisy in the downsampled dataset.

**Supplementary Fig. S7**. Runtimes for 4 bioinformatics preprocessing steps: trimming, aligning (bwa mem algorithm), postalignment filtering, and converting BAM to BED files.

## Abbreviations

AUC: area under the curve (AUC); BWA: Burrows–Wheeler aligner; cfDNA: cell-free DNA; CNA: copy number aberration; ctDNA: circulating tumor DNA; DHS: DNase hypersensitivity site.

## Supplementary Material

giaf139_Supplemental_File

giaf139_Authors_Response_To_Reviewer_Comments_Original_Submission

giaf139_GIGA-D-25-00297_Original_Submission

giaf139_GIGA-D-25-00297_Revision_1

giaf139_Reviewer_1_Report_Original_Submission Wei Zhang -- 8/17/2025

## Data Availability

The dataset supporting the results of this article is available in the European Genome-Phenome Archive under the accession EGAD50000000213 [50]. We used the following genome builds: hg19 [51], hs37d5 [52], hg38 (Homo_sapiens_assembly38.fasta file) [53], and hg38 without alternative contigs (GCA_000001405.15_GRCh38_no_alt_analysis_set.fna.gz) [54].
