# Supplementary material for: The effects of bioinformatics preprocessing on cell-free DNA fragment analysis
Source: Gigascience. 2025 Oct 30;14:giaf139. doi: 10.1093/gigascience/giaf139 (PMC12720587; doi:10.1093/gigascience/giaf139)
Supplement: giaf139_Supplemental_File [file giaf139_supplemental_file.pdf]

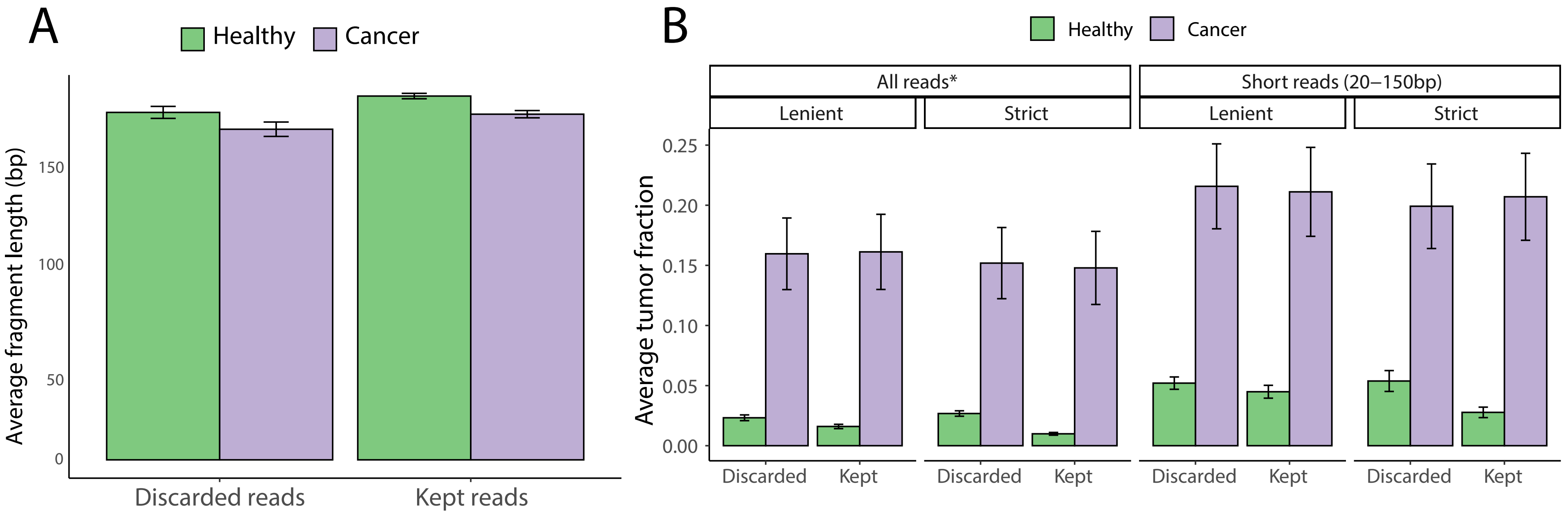

Supplementary Figure 1. A) The average fragment lengths of discarded reads are shorter than those of kept reads. B) Tumor fractions calculated with ichorCNA were compared across different preprocessing settings for both kept and discarded reads, considering all reads as well as only short reads. It was observed that discarded reads do not show enrichment in tumor DNA, as their tumor fractions do not increase compared to those of cancer samples with kept reads. *downsampled to match the coverage of BAM files containing only short reads.

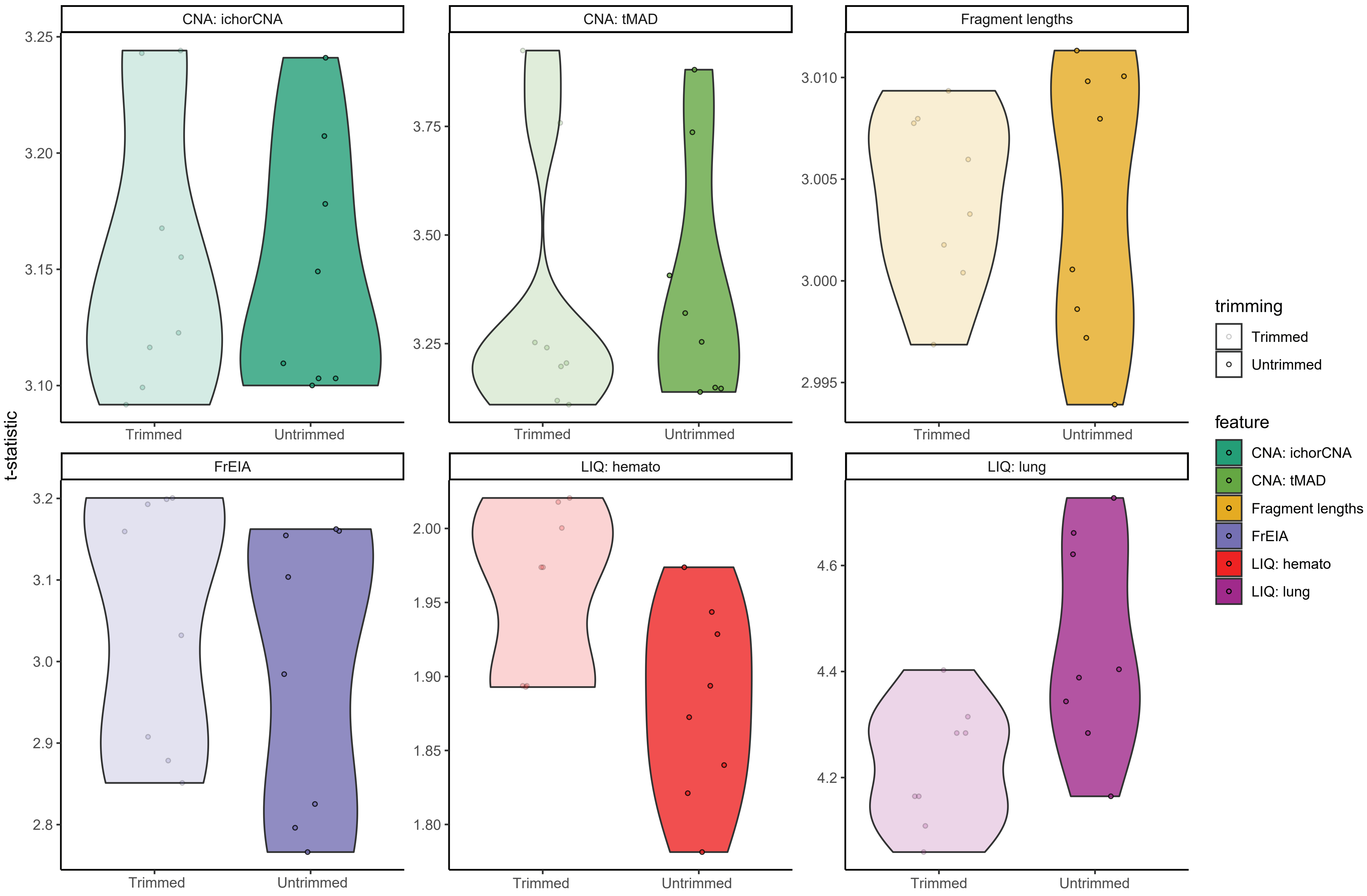

Supplementary Figure 2. The effect of trimming on distinguishing healthy and cancer samples. t-statistics from t-tests comparing healthy and cancer samples were calculated for various features, including tumor fraction estimated by ichorCNA, tMAD score, average fragment length, hematopoietic and cell type signatures from LIQUORICE, and normalized coverage. Each data point represents a unique preprocessing setting combination (16 per feature).

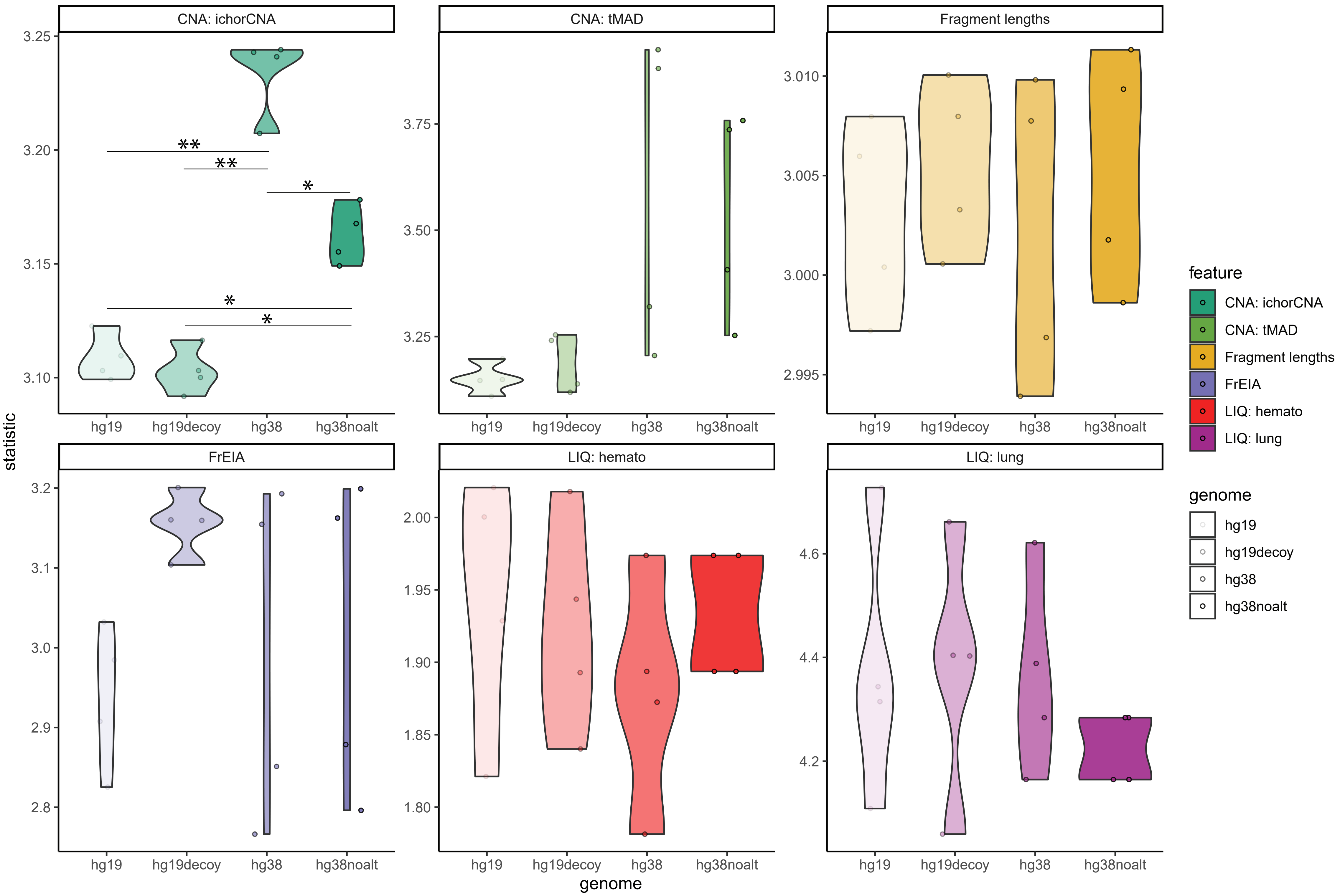

Supplementary Figure 3. The effect of genome build on distinguishing healthy and cancer samples. t-statistics from t-tests comparing healthy and cancer samples were calculated for multiple features, including tumor fraction estimated by ichorCNA, tMAD score, average fragment length, hematopoietic and cell type signatures from LIQUORICE, and normalized coverage. Each data point represents a unique preprocessing setting combination (16 per feature). Significance values correspond to results of pairwise t-tests, corrected for multiple hypothesis testing using the Bonferroni method.

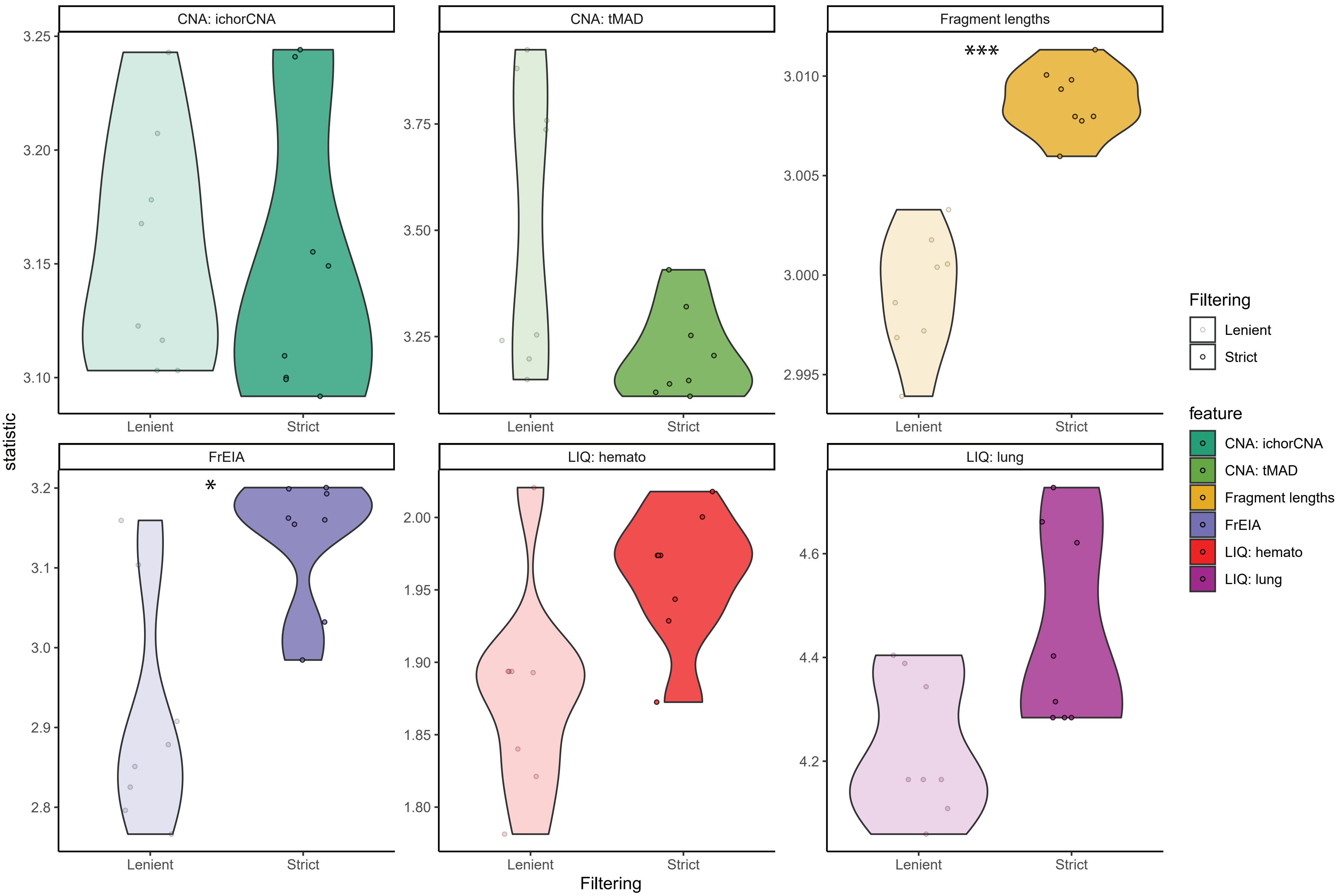

Supplementary Figure 4. The effect of GC-bias correction on distinguishing healthy and cancer samples. t-statistics from t-tests comparing healthy and cancer samples were calculated for several features, including tumor fraction estimated by ichorCNA, tMAD score, average fragment length, hematopoietic and cell type signatures from LIQUORICE, and normalized coverage. Each data point represents a unique preprocessing setting combination (16 per feature).i Sgnificance values correspond to results of pairwise t-tests, corrected for multiple hypothesis testing using the Bonferroni method.

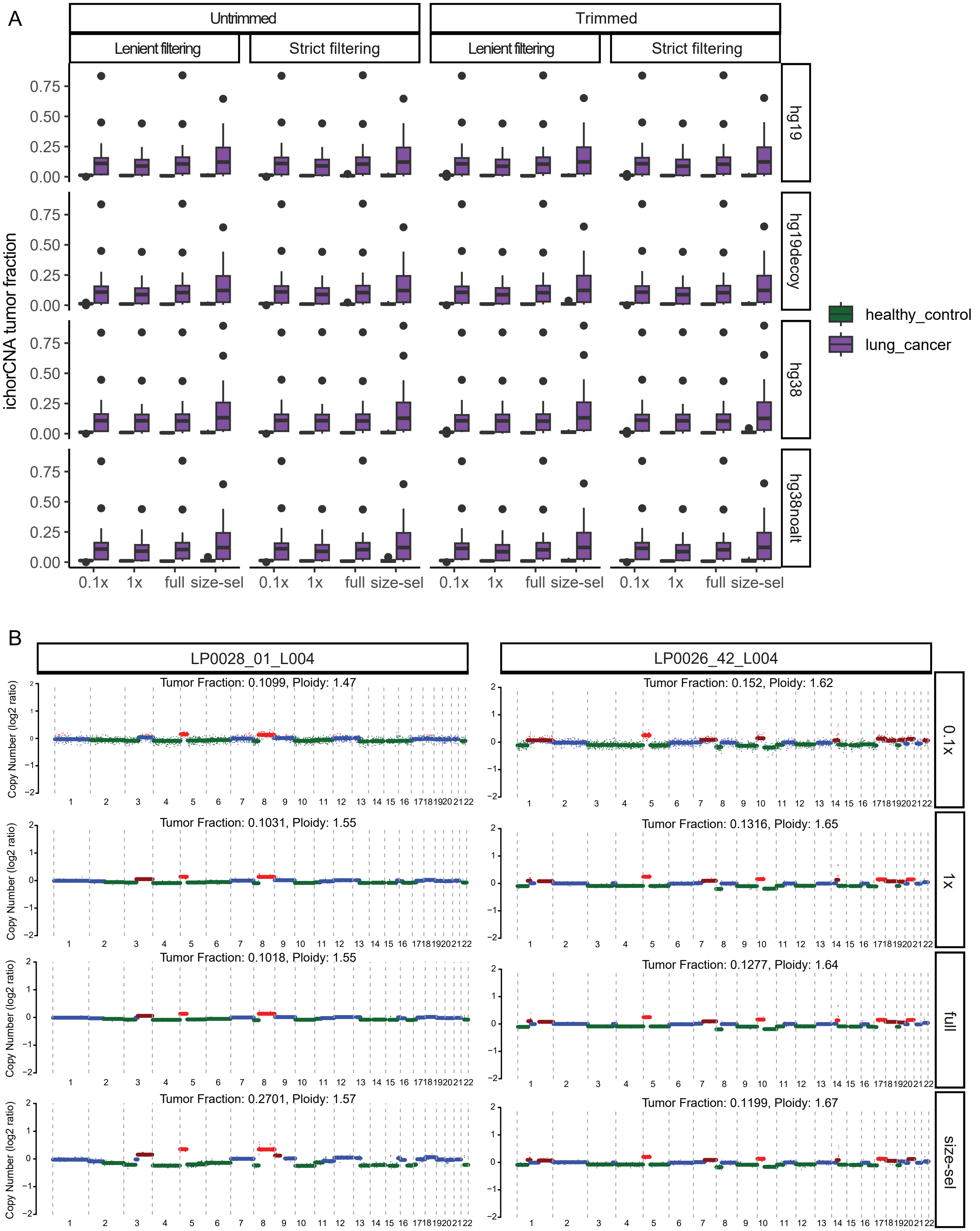

Supplementary Figure 5. The effects of downsampling and size-selection on copy-number analysis. A) tumor fraction estimates output by ichorCNA for healthy (green) and cancer samples (purple). The boxes depict interquartile ranges. For each setting n(healthy)=20 and n(cancer)=20. B) Selected copy number plots from two cancer patients, generated with ichorCNA. LP0028_01_L004's copy number analysis was improved by selecting for short fragments (<150 bp) whereas LP0026_42_L004's was not.

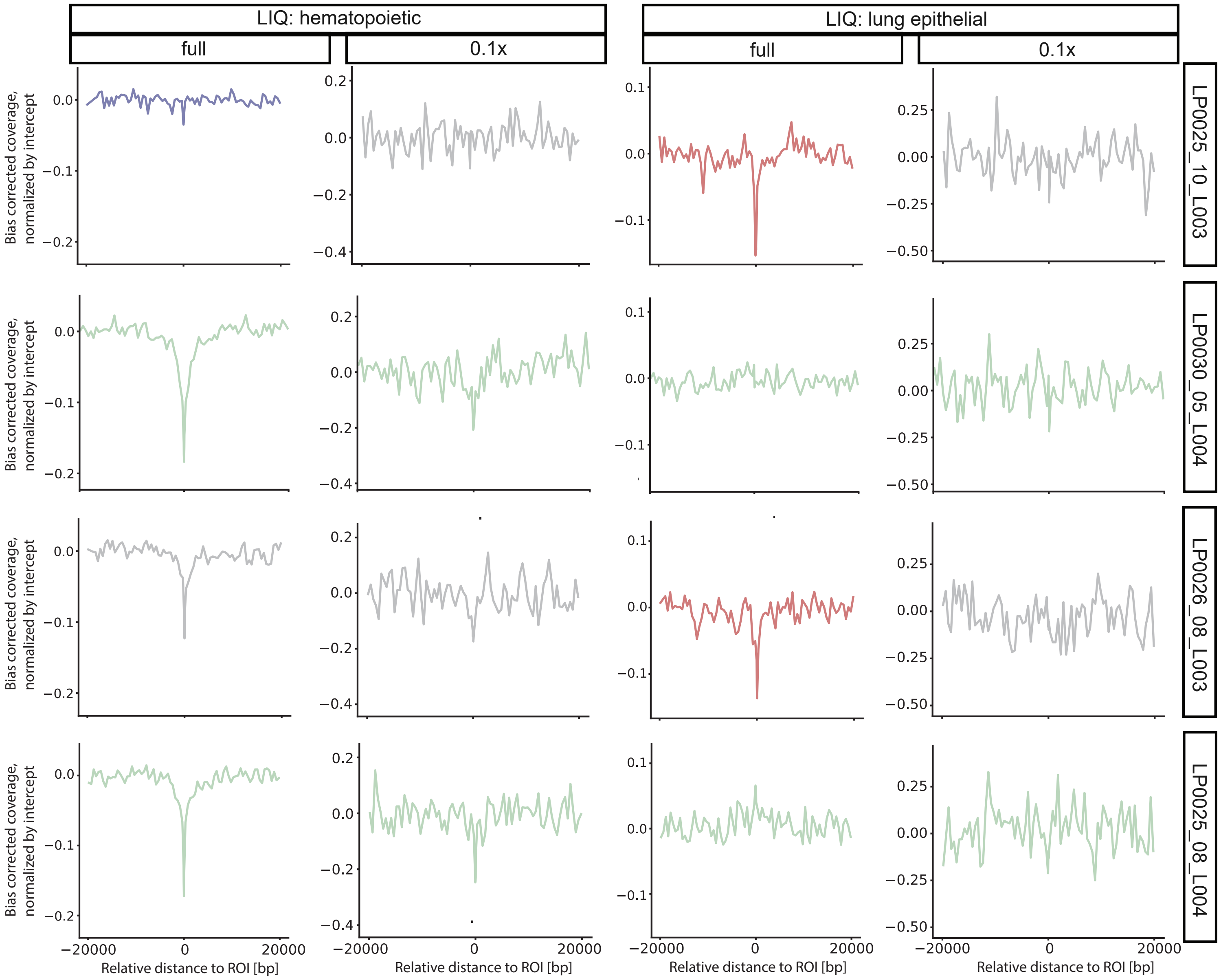

Supplementary Figure 6. The effects of downsampling on nucleosome footprint analysis with LIQUORICE. Four patients' LIQUORICE plots are shown. The left columns show coverage values at hematopoietic-specific promoter regions and the right columns at lung epithelial-specific promoter regions. Green: healthy control. Blue: cancer sample's dip in coverage significantly less than in controls. Red: cancer sample's dip in coverage significantly more than in controls. Grey: cancer sample not significantly different from controls. The plots show that cell-type signatures are clear in the full coverage data, but are very noisy in the downsampled dataset.

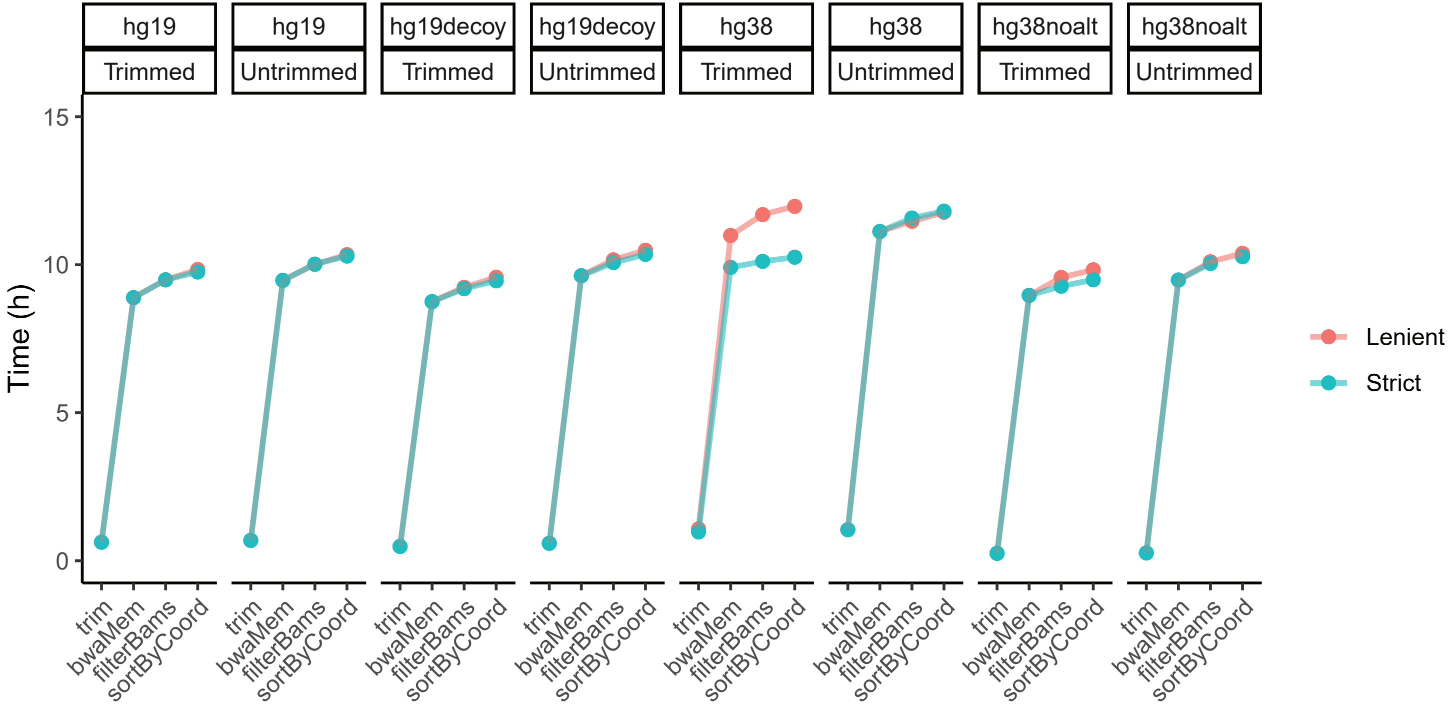

Supplementary Figure 7. Runtimes for four bioinformatics preprocessing steps: trimming, aligning (bwa mem algorithm), post-alignment filtering and converting BAM to BED files.
