# Supplementary material for: The effects of bioinformatics preprocessing on cell-free DNA fragment analysis
Source: Gigascience. 2025 Oct 30;14:giaf139. doi: 10.1093/gigascience/giaf139 (PMC12720587; doi:10.1093/gigascience/giaf139)
Supplement: giaf139_GIGA-D-25-00297_Original_Submission [file giaf139_giga-d-25-00297_original_submission.pdf]

GigaScience

The effects of bioinformatics preprocessing on cell-free DNA fragment analysis

--Manuscript Draft--

**Manuscript Number:**	GIGA-D-25-00297
**Full Title:**	The effects of bioinformatics preprocessing on cell-free DNA fragment analysis
**Article Type:**	Research
**Funding Information:**	Candoc Grant of the University of Zurich (FK-22-089) — Dr Ivna Ivanković<br>Forschungskredit Postdoc Grant of the University of Zurich (FK-20-103) — Dr. Zsolt Balázs<br>KWF Kankerbestrijding (KWF-12822) — Dr Florent Mouliere
**Abstract:**	Background<br><br>While cell-free DNA (cfDNA) is a promising biomarker for cancer diagnosis and monitoring, there is limited agreement on optimal cfDNA collection and extraction protocols as well as analysis pipelines of the corresponding cfDNA sequencing data. In this paper, we address the latter by studying the effect of various bioinformatics preprocessing choices on derived genetic and epigenetic cfDNA features and study how observed feature differences influence the downstream task of separating between healthy and cancer cfDNA samples.<br><br>Results<br><br>Using low-pass whole-genome cfDNA sequencing data from 20 lung cancer and 20 healthy samples, we assessed the influence of various preprocessing settings such a read trimming, filtering of secondary alignments and choice of genome build as well as practices such as downsampling or selecting for short fragment on derived cfDNA features including cfDNA fragment size, fragment end motifs, copy number alterations, and nucleosome footprints. Our results demonstrate that the analyzed features are robust to common preprocessing choices, but exhibit variable sensitivity to sequencing coverage. Fragment length statistics and end motifs are the least affected by low coverages, whereas nucleosome footprint analysis is very sensitive to it. Our findings confirm that selecting for shorter fragments, enhances cancer-specific signals, however, by removing data, also reduces signals in general. Interestingly, we find that fragment end motif analysis benefits the most from in silico size selection. We also observe that the filtering of low-quality and secondary alignments and choice of genome build result in slight improvements in cancer classification performance based on nucleosome coverage and copy number features.<br><br>Conclusions<br><br>Altogether, we conclude that cfDNA analysis is minimally affected by different bioinformatics preprocessing settings, however we describe some synergistic effects between analytical approaches, which can be leveraged to improve cancer detection.
**Corresponding Author:**	Michael Krauthammer<br>University of Zurich: Universitat Zurich<br>Zurich, Zurich SWITZERLAND
**Corresponding Author Secondary Information:**	
**Corresponding Author's Institution:**	University of Zurich: Universitat Zurich
**Corresponding Author's Secondary Institution:**	
**First Author:**	Ivna Ivanković, PhD
**First Author Secondary Information:**	

Powered by Editorial Manager® and ProduXion Manager® from Aries Systems Corporation

Order of Authors:	Ivna Ivanković, PhD
	Zsolt Balázs, PhD, MD
	Todor Gitchev, MSc
	Cécile Trottet, MSc
	Norbert Moldovan, PhD
	Idris Bahce, PhD MD
	Florent Mouliere, PhD
	Michael Krauthammer, PhD MD
Order of Authors Secondary Information:	
Additional Information:	
**Question**	**Response**
Are you submitting this manuscript to a special series or article collection?	No
**Experimental design and statistics**<br><br>Full details of the experimental design and statistical methods used should be given in the Methods section, as detailed in our Minimum Standards Reporting Checklist. Information essential to interpreting the data presented should be made available in the figure legends.<br><br>Have you included all the information requested in your manuscript?	Yes
**Resources**<br><br>A description of all resources used, including antibodies, cell lines, animals and software tools, with enough information to allow them to be uniquely identified, should be included in the Methods section. Authors are strongly encouraged to cite Research Resource Identifiers (RRIDs) for antibodies, model organisms and tools, where possible.<br><br>Have you included the information requested as detailed in our Minimum Standards Reporting Checklist?	Yes
**Availability of data and materials**	Yes

Powered by Editorial Manager® and ProduXion Manager® from Aries Systems Corporation

All datasets and code on which the conclusions of the paper rely must be either included in your submission or deposited in publicly available repositories (where available and ethically appropriate), referencing such data using a unique identifier in the references and in the "Availability of Data and Materials" section of your manuscript.<br><br>Have you have met the above requirement as detailed in our Minimum Standards Reporting Checklist?	
GigaScience has policies and guidelines in place for the use of generative AI-writing tools such as ChatGPT. If you have used such writing tools to assist with writing the manuscript this must be declared and cited in the text. Authors should not list AI-writing tools and other AI-assisted technologies as an author or co-author and should acknowledge that they are fully responsible for text generated or refined by AI-writing tools.<p><br><br>A summary of use (particularly in the introduction or among methods) needs to be included at the end of the paper, and the outputs should also be included as a supplementary file hosted in GigaDB or other open repositories. Please read our guidelines for more information. <p><br><br>By submitting to GigaScience, you are aware of the journal's AI-writing tools policy, and if you have declared use of such tools below, you have acknowledged this where appropriate in your manuscript and have made a summary of use and outputs available. <p><br><b>AI-assisted writing tools have been used in the preparation of this manuscript?	No

Powered by Editorial Manager® and ProduXion Manager® from Aries Systems Corporation

Manuscript

Click here to access/download;Manuscript;preprocessing_manuscript_text.p

The effects of bioinformatics preprocessing on cell-free DNA fragment analysis

Ivna Ivanković[1,2†], Zsolt Balázs[1,2†], Todor Gitchev[1,2], Cécile Trottet[1,2], Norbert Moldovan[3,4], Idris Bahce[5], Florent Mouliere[3,4,6,*], Michael Krauthammer[1,2,*]

[1] Department of Quantitative Biomedicine, University of Zurich, Switzerland

[2] University Hospital Zurich University Hospital Zurich, Switzerland

[3] Amsterdam UMC, Vrije Universiteit Amsterdam, Department of Pathology, Cancer Centre Amsterdam, Amsterdam, The Netherlands

[4] Cancer Center Amsterdam, Imaging and Biomarkers, Amsterdam, The Netherlands

[5] Amsterdam UMC, Vrije Universiteit Amsterdam, Department of Pulmonology, Cancer Centre Amsterdam, Amsterdam, The Netherlands

[6] Cancer Research UK National Biomarker Centre, University of Manchester, Manchester, United Kingdom

[†] These authors contributed equally

* corresponding authors

Abstract

Background: While cell-free DNA (cfDNA) is a promising biomarker for cancer diagnosis and monitoring, there is limited agreement on optimal cfDNA collection and extraction protocols as well as analysis pipelines of the corresponding cfDNA sequencing data. In this paper, we address the latter by studying the effect of various bioinformatics preprocessing choices on derived genetic and epigenetic cfDNA features and study how observed feature differences influence the downstream task of separating between healthy and cancer cfDNA samples.

Results: Using low-pass whole-genome cfDNA sequencing data from 20 lung cancer and 20 healthy samples, we assessed the influence of various preprocessing settings such a read trimming, filtering of secondary alignments and choice of genome build as well as practices such

as downsampling or selecting for short fragment on derived cfDNA features including cfDNA fragment size, fragment end motifs, copy number alterations, and nucleosome footprints. Our results demonstrate that the analyzed features are robust to common preprocessing choices, but exhibit variable sensitivity to sequencing coverage. Fragment length statistics and end motifs are the least affected by low coverages, whereas nucleosome footprint analysis is very sensitive to it. Our findings confirm that selecting for shorter fragments, enhances cancer-specific signals, however, by removing data, also reduces signals in general. Interestingly, we find that fragment end motif analysis benefits the most from *in silico* size selection. We also observe that the filtering of low-quality and secondary alignments and choice of genome build result in slight improvements in cancer classification performance based on nucleosome coverage and copy number features.

Conclusions: Altogether, we conclude that cfDNA analysis is minimally affected by different bioinformatics preprocessing settings, however we describe some synergistic effects between analytical approaches, which can be leveraged to improve cancer detection.

Introduction

Cell-free DNA (cfDNA) fragments are released into the bloodstream during cell death and carry genetic and epigenetic information from their cells of origin [1–3]. The analysis of cfDNA emerged as a valuable diagnostic tool in cancer detection and screening, since sample collection is minimally invasive and cfDNA fragmentation patterns provide valuable clinical insights [4]. In healthy individuals, cfDNA is mainly derived from hematopoietic cells, while in patients with cancer, a fraction of this cfDNA is released from the tumor cells, known as circulating tumor DNA (ctDNA) [5,6].

The use of next generation sequencing technologies has advanced the investigation of fragmentomic and genomic profiles of cfDNA-based liquid biopsies [7]. A substantial portion of cfDNA research is directed towards identifying and studying characteristics of cfDNA through low-coverage sequencing data, offering a cost-effective approach for cancer detection, monitoring disease progression and treatment response [8]. cfDNA fragments derived from tumors are generally shorter than those originating from hematopoietic cells [9,10]. Estimating copy number aberrations from low-coverage sequencing data provides a cost-effective solution for calculating the tumor fraction (portion of ctDNA in the total cfDNA). Furthermore, cfDNA fragmentation is non-random and can differ depending on the epigenetic environment and gene expression in the cell types of origin [11,12]. Non-random fragmentation affects not only the genomic location of the cfDNA fragments but also the sequences at the end of fragments [13–15]. These distinctive features, including fragment length, fragment end motif, nucleosome positioning and coverage at specific genomic regions, not only facilitate the differentiation between diseased and healthy individuals but also offer information about the cell type of origin and thus have a great potential to serve as cfDNA biomarkers.

While the clinical benefits of studying cfDNA have been established, a systematic comparison of both experimental and computational methods is essential to develop and standardize best practices in cfDNA analysis. Efforts towards standardizing cfDNA measurements have been made, by comparing the DNA extraction kits [16] and studying the effects of preanalytical and physiological variables on cfDNA fragmentation [17]. Even though many software tools have been developed for the analysis of cfDNA genetic and fragmentomic features (a list of such tools is collected on the NucPosDB website [18]), the effects of various bioinformatics preprocessing approaches on these features have not been thoroughly examined to this date.

Prior works on tissue biopsy highlighted the effects of bioinformatics preprocessing on the analysis of sequencing data, such as variant calling, gene expression analysis, and copy number variation analysis [19–21]. Accurate alignment in variant calling analyses hinges on the choice of the reference genome, read trimming and post-alignment filtering, as mismatches can lead to false variant calls [22,23]. A recent paper [24] found that using different genome aligners and trimming options on cfDNA fragment length and end motifs. These findings further highlight that the careful optimization of these preprocessing steps is imperative to ensure robust and meaningful biological insights from cfDNA genome-wide sequencing.

Here, we systematically evaluate the effects of bioinformatics preprocessing on the analysis of whole-genome cfDNA sequencing data. We present cfDNA-Flow, a modular bioinformatics pipeline offering a range of preprocessing options. Using cfDNA-Flow, we comprehensively evaluate the effects of preprocessing settings on cfDNA sequencing data. We evaluate our results according to how each modality differs between healthy and cancer cfDNA samples, and whether they impact the performance of liquid biopsy assays.

Data Description

Patient recruitment and sample processing

Patient and healthy individual samples were recruited following informed consent via the Liquid Biopsy Center at the Amsterdam UMC, location VUmc and location AMC (study approved by the Amsterdam UMC ethics board, METC U2019_035). Blood samples were processed as previously described [25]. In brief, blood was collected in EDTA tubes and processed via a double centrifugation protocol (900g for 15 min, 2500g for 10 min) and supernatant plasma stored at - 80°C. DNA was isolated using the QIAsymphony DSP Circulating Nucleic Acids kit (QIAgen). cfDNA was quantified using a cfDNA kit and a Tapestation 4200 system (Agilent), Indexed sequencing libraries were prepared using 1-10 ng of DNA and the ThruPLEX Plasma-seq kit (Takara). Libraries were pooled in equimolar amounts and sequenced to >1x coverage on a NovaSeq 6000 (Illumina) generating 150 bp paired-end reads from a S4 flowcell.

Analyses

Overview

We built a pipeline for reproducible analysis using a scalable bioinformatics workflow engine, Snakemake [26], which enabled us to preprocess samples testing different settings (Figure 1) and different post-alignment filtering options and extract biologically relevant features. We applied our pipeline to a cohort of 20 healthy and 20 late-stage lung cancer samples (18/20 stage IV) (Figure 1). Their tumor fraction, estimated based on the amplitude of copy number aberrations using ichorCNA, ranged between 0.6% and 84%, with the mean of 15% and a detection threshold of 3%. Raw read coverage ranged between 0.04x to 10.9x, with the mean of 2.5x. From all

samples, we extracted six features covering four distinct modalities which have previously been used to classify tumor and healthy plasma cfDNA samples: the (1) cfDNA fragment length, (2) ichorCNA tumor fraction and (3) tMAD score based on copy number alterations, (4) fragment end motifs and nucleosome coverage over (5) blood-cell specific and (6) cancer-specific Dnase hypersensitivity sites (DHSs) [4,10,27,28].

The effects of bioinformatics preprocessing on fragment count statistics

We investigated reads removed during the following bioinformatics preprocessing steps: quality trimming, alignment, post-alignment filtering. We found that trimming and discarding reads that were shorter than 50 bp after trimming reduced the number of reads by 2%. The read count loss between trimmed and non-trimmed files was less prominent after alignment and post-alignment filtering, because many reads that would have needed extensive trimming, were either not mapped or mapped ambiguously. The choice of genome build did not affect the read count substantially. We tested 2 post-alignment filtering options: lenient filtering, where we only removed unmapped and duplicate reads and strict filtering settings, where, in addition, we removed low-quality, indel-containing reads, and any read that had a secondary alignment. On average, discarded fragments were shorter than kept fragments (Supplementary Figure 1A). It has been observed that cancer-derived cfDNA fragments tended to be short [10], however the discarded reads were not enriched in tumor-derived cfDNA, as copy number analysis-based tumor fraction estimates were not increased when analyzing the discarded reads (Supplementary Figure 1B). Moreover, the reads discarded by strict filtering even showed a decreased tumor fraction estimate for the cancer samples and an increased estimate for the healthy samples, which confirms that these discarded reads are not helpful in distinguishing cancer and healthy samples.

The effects of bioinformatics preprocessing on genetic and epigenetic cfDNA features

All six of the studied features appeared robust to the 16 examined preprocessing settings, with no statistical difference in their mean values observed (ANOVA, $p = 1$). The studied features exhibited low variance, with lung epithelial cell type signature, fragment end motifs, and CNA-derived features displaying slightly higher variance than fragment lengths and hematopoietic cell type signature (Figure 2A). Most of the observed variance was explained by the filtering and the choice of the reference genome, while trimming had the least effect (Figure 2B).

Even though we did not observe any significant difference in the means of features derived by 16 studied preprocessing settings, noticeable patterns emerged after correlating the values of a feature calculated using one preprocessing setting with those calculated using every other preprocessing setting (Figure 2C). Features derived from copy number aberrations (CNA), such as tumor fraction and nucleosome footprints, indicated possible dependence on the choice of reference genome, and fragment end motifs' FrEIA scores showed higher correlation if the same alignment filtering was applied.

The effects of bioinformatics preprocessing on classifying cancer and healthy samples

We performed t-tests to compare each feature's ability to differentiate cancer and healthy samples across 16 studied preprocessing settings (Figure 3A). Interestingly, the greatest difference between the means of cancer and healthy samples was observed in lung epithelial cell-type signatures and the least in the hematopoietic cell-type signatures, across all settings. Copy number analyses using ichorCNA and tMAD achieved similarly good separation to using fragment length averages and fragment end motifs. Then, we classified samples based on each feature individually (Figure 3B) to further investigate the effects of preprocessing settings on studied

features and the distinction between the healthy and cancer samples. Tumor fraction calculated using ichorCNA showed the highest, whereas hematopoietic cell signature showed lowest AUC scores. The effects of the investigated preprocessing decisions (trimming, reference genome choice, alignment filtering) on distinguishing healthy and cancer samples are detailed in the paragraphs below.

Trimming: Read trimming had no impact on the observed average fragment lengths features derived from copy number aberrations, and surprisingly, not even on fragment end motifs (Figure 3A). Nucleosome footprint analysis was slightly but not significantly affected by trimming the reads (lung p.adj = 0.1056 and hematopoietic cell signatures p.adj = 0.1554) (Supplementary Figure 2).

Reference genome choice: The choice of reference genome had no impact on the fragment lengths nor cell-type specific nucleosome signatures (Figure 3A). CNA-derived features, especially CNA calculated by ichorCNA, were significantly affected by the choice of reference genome, showing better performance in distinguishing between healthy and cancer samples in the hg38 and hg38noalt genomes compared to the hg19 and hg19decoy genomes (Figure 3A, Supplementary Figure 3). Lung epithelial signatures differed slightly (not significantly) more between healthy and lung cancer samples when using the hg19 or hg19decoy genome builds. However, it has to be noted, that to determine the cell-type specific nucleosome footprints, we used a list of DHSs that was annotated on the hg19 genome and coordinates were converted to the hg38 build using Liftover (see Methods).

Alignment Filtering: Strict filtering increased the differences between healthy and cancer patients when measuring fragment lengths (p.adj=0.00004) and fragment end motifs (p.adj= 0.0162, Figure 3A, Supplementary Figure 5). Furthermore, nucleosome footprint analysis achieved slightly, albeit not significantly (p.adj = 0.1104 for lung epithelial, and p.adj=0.1134 for hematopoietic signatures) better distinction of the healthy and cancer cohort when applying strict alignment filtering.

The effects of downsampling and size selection on classifying cancer and healthy samples

Best practices often call for downsampling the input samples to the same coverage in order to normalize for signal-to-noise ratio which might bias downstream inference. In order to test the effects of downsampling, and also the coverage-dependence of the tested features, we downsampled our dataset to uniformly 0.1x and 1x coverage and compared the results to those of the non-uniform full coverage dataset. As short fragments have been shown to be more likely originate from cancer cells [29], we also performed *in silico* selection of short fragments (<150 bp) and analyzed the same features.

Downsampling: While features such as fragment length averages and fragment end motifs were robust to downsampling, copy number analysis both using ichorCNA and tMAD became noisier at lower coverages (Supplementary Figure 5B), although cancer-healthy classification remained accurate even at 0.1x coverage, consistent with earlier publications [30] (Figure 4). Nucleosome footprints analyzed by LIQUORICE were very sensitive to coverage (Supplementary Figure 6), with AUC values only slightly better than random chance (Figure 4).

Size selection: As size selection in our dataset results in very low coverage data (<0.1), we could not evaluate the effects of size selection on nucleosome footprints. Investigating the effects of size selection on copy-number analysis, we found that while the average tumor fraction estimates (Figure 4) increased upon size selection, the accuracy of cancer-healthy classification did not. This is because in some cancer samples, size selection did not increase the tumor fraction estimates, however, the very low coverage of the data, increased noise. Most interestingly however, we found that cancer-healthy classification based on fragment end motifs was substantially improved by size selection. We attribute this to the selective enrichment of cancer-specific fragment end motifs which was not hindered by the very low coverage of the dataset, as fragment end motif analysis is generally robust to coverage,

Computational resources and runtimes

Computation runtimes for the preprocessing steps ranged between 9.5 and 14 hours (median = 11.4 hours) for 40 analyzed samples (Supplementary Figure 7). Peak RAM usage was observed during the alignment step where it exceeded 16-18GB per core. Trimmed and untrimmed reads showed no significant CPU usage difference likely due to high-quality raw reads.

Discussion

We built a versatile bioinformatic pipeline and benchmarked commonly used bioinformatics preprocessing settings on a low-pass whole-genome sequencing dataset containing plasma cfDNA of 20 healthy individuals and 20 patients with lung cancer. We derived genetic, fragmentomic and epigenetic features from the cfDNA data and assessed the effects of the preprocessing settings on those features. Our principal findings underscore the robustness of studied genetic, epigenetic and fragmentomic features across the 16 preprocessing settings investigated. We detected no statistically significant differences in the mean values of these features across different preprocessing settings.

Although we found that the majority of the examined preprocessing settings did not substantially change the outcome of the analyses, we found a slight advantage to using recent genome builds (hg38 vs hg19) when performing copy number analysis. We believe that the better curated genome build allowed for fewer misaligned reads, which ultimately led to a better performance of these tools with regards to classifying cancer. However, as not every annotation is available for all genome builds, users may still be limited to the use of genome builds which have the annotation they are interested in. We found that nucleosome footprint analysis which relied on pre-existing

annotation, distinguished healthy and cancer samples better when the annotation matched the genome build of the annotation. This is possibly caused by inaccurate conversion between genome build coordinates [31]. Interestingly, we did not observe a difference in aligning to genomes with decoy or alternative contigs and more minimalist genome builds of the same version (e.g. hg38 vs hg38noalt). Further, we observed that strict alignment filtering (i.e. removing secondary alignments and reads with poor mapping quality or that align with indels) improved the distinction of patients with lung cancer and healthy individuals when analyzing fragment lengths, fragment end motifs and, to a lesser degree, nucleosome footprints.

By performing downsampling, we confirmed previous publications' conclusions that (1) fragment length-, and fragment end motif-based analyses are not affected by sequencing coverage, that (2) accurate copy number analysis calling requires >1x coverage, but cancer-healthy classification is feasible with 0.1x coverage data at least with late-stage cancer cohorts and finally that (3) nucleosome footprinting requires high (>1x) coverage input. Investigating the effects of size selection, we found that enriching for cancer-specific fragments and greatly reducing the coverage creates a trade-off for copy-number analysis that has to be taken into consideration. Importantly however, fragment end motif analysis is not sensitive to coverage but benefits from the enrichment of cancer specific fragments and therefore can only benefit from selecting for shorter fragments *in silico*.

Limitations of our study include that we have analyzed a relatively small dataset and that the cancer samples were of late-stage lung cancer patients. We focused our analyses on late-stage cancer samples, with clear cancer-specific alterations, in order to provide sufficient signals for analysis of various features, as early-stage cancers are often undetectable from liquid biopsy data [32,33]. Furthermore, the focus of our analysis was sWGS which can output CNVs and fragmentation data. Our analysis therefore did not include methylation related or mutation related data which are also among the most intensively investigated areas of liquid biopsy [34–36]. However, we emphasize that there is a lack of computational benchmarking on mutation and

methylation analyses and those are likely affected differently by preprocessing than the features analyzed in our study. Further research in the field of DNA methylation and mutations could expand and complement our findings on CNVs and fragmentation data.

In conclusion, we developed a modular reproducible cfDNA-sequencing preprocessing workflow called cfDNA-Flow and evaluated several bioinformatics preprocessing settings for the analysis of low-pass whole-genome cfDNA sequencing data. Our findings show that most preprocessing settings have little impact on downstream analysis with only strict alignment filtering improving the detection of cancer samples. In general, our recommendations are to use strict alignment filtering to remove any ambiguous alignments and to use genome builds with the appropriate annotation for any downstream tasks.

Methods

Preprocessing

For computing, we used 16 CPUs and 18 and 16 GB RAM per core for the alignment and the downstream analysis, respectively.

Read trimming

In order to test the effects of trimming on the analysis of cfDNA sequencing data, trimming was performed using the skewer [37] tool (v0.2.2), an efficient trimmer for paired-end reads. We explored two settings: 1) no read quality filtering nor trimming, 2) read quality filtering and trimming with the following values: lowest mean quality of the read (*Q*) allowed before trimming was set to 30, minimum length allowed after trimming (*l*) to 50 base pairs and trimming the 3' end of the reads until the quality (*q*) of at least 35 was reached.

Reference genome choice and mapping

We carried out our analyses on sequencing reads aligned to four different human reference genome builds: hg19, hs37d5, hg38 and hg38 without alternative contigs (for download links see the Data availability section). Mapping against the reference genome was performed using Burrows-Wheeler Aligner (BWA) software package's [38] (v0.7.17-r1188) bwa-mem algorithm. Duplicates are marked using Picard tools (v2.27.1) from The Genome Analysis Toolkit [39] (v4.2.6.1).

Quality control

The quality of raw FASTQ files were assessed using FastQC (v0.11.9) and alignment summary metrics are calculated using the Picard toolkit. A MultiQC [40] (v1.13.dev0) report is generated both for FastQC and Picard alignment metrics.

Post-alignment filtering

To test the effects of post alignment filtering, we explored the following post-alignment filtering options: strict filtering (removing unmapped reads, reads that are or have a secondary alignment, reads with mapping quality <30 and reads with deletions or insertions in them) and lenient filtering (removing only unmapped reads).

Analyzing the effects of read filtering

To analyze properties of reads that were filtered out during trimming and alignment filtering, we studied and compared discarded and kept reads. We used the 'diff' option from the bamUtil (v1.0.15) [41] repository to create BAM files containing only discarded reads by finding a difference between the unfiltered BAM file and the final BAM files after preprocessing. We downsampled preprocessed BAM files containing kept reads to the coverage of newly generated

BAM files containing the reads which were filtered out during the analysis. Subsequently, we calculated tumor fraction using ichorCNA [27] for kept reads and discarded reads both selecting for short fragments (20-150 bp) and keeping fragments of all sizes. Additionally, we calculated and compared the fragment lengths in BAM files with discarded and kept reads.

Feature extraction

Fragment length features

We calculated mean, median and standard deviation values for fragments sized 100 to 220 base pairs (bp), which correspond to the mononucleosomal size range. The size range corresponds to fragments originating predominantly from mononucleosomes. We also calculated the frequencies of cfDNA fragment sizes ranging from 70bp to 1000bp in 10bp bins.

Copy number analysis

We used copy number analysis tools, ichorCNA (v0.2.0) [27] and tMAD [10], to estimate copy number changes and tumor fraction. We created a panel of normals using the 20 healthy samples and ran ichorCNA assuming diploid state and copy numbers up to 3 with an a priori estimates of normal fraction set to: 0.5, 0.9, 0.95, 0.99, 0.995 and 0.999.

Fragment end motifs

We used the FrEIA tool [15] with default settings. However, preprocessing steps (such as trimming) otherwise performed by FrEIA were skipped, in order to allow for the comparison of results between the different preprocessing approaches. Cancer and control fragment end motifs were determined once, using the original dataset.

Differential coverage analysis over DNase hypersensitivity sites

We examined differential coverage over DNase hypersensitivity sites (DHSs) in hematopoietic and small airway epithelial cells (SAEC) using the LIQUORICE software (v0.5.4) [42]. Region sets specific for cell types were defined using cell-type specific clusters of DHSs based on data from the University of Washington [43] and Duke ENCODE groups [44]. The downloaded regions were coordinates on the hg19 genome, which were converted to hg38 coordinates using Liftover [45].LIQUORICE queries the average coverage and models the dip in coverage over the specified region sets presuming the data can be best described as a sum of three Gaussian distributions. The calculated dip depth values are compared to a control group (healthy cohort) and z-scaled. We used the z-scaled coverage dip depth values as cell-type specific signatures.

Downsampling and size selection

Both downsampling and size selection were performed using samtools view [46,47]. For downsampling, the coverage of a given was calculated and samtools view's subsampling proportion was calculated to achieve 0.1x and 1x coverages. Size selection was performed only retaining fragments shorter than 150 bp.

Reproducibility and scalability

We developed cfDNA-Flow pipeline using software for scalable and reproducible analysis, Snakemake [26], following best code-development guidelines. Snakemake enables the user to use the pipeline on single-core workstations or compute clusters without modifying the code. We also provide a Singularity [48] recipe for building a container containing all needed packages and tools to run all pipeline steps. The architecture also allows cloning and running the pipeline code using tools from the Singularity container. The code is available at https://github.com/uzh-dqbm-cmi/cfDNA-Flow.

Ethics, consent and permissions

We report on data collected for the study METC U2019_035, which was approved by the Amsterdam UMC ethics board. Informed consent was obtained from all study participants.

Code availability

The code used to generate figures with the underlying data can be found at https://github.com/uzh-dqbm-cmi/cfDNA-Flow_paper. The source code of the pipeline can be found at https://github.com/uzh-dqbm-cmi/cfDNA-Flow and is available with a GPL-v3 licence.

Data availability

The dataset supporting the results of this article is available in the European Genome-Phenome Archive under the accession EGAD50000000213. The genome build's fasta files were downloaded from: hg19:https://storage.cloud.google.com/genomics-public-data/references/b37/Homo_sapiens_assembly19.fasta.gz, hs37d5: http://ftp.1000genomes.ebi.ac.uk/vol1/ftp/technical/reference/phase2_reference_assembly_sequence/hs37d5.fa.gz, hg38 (Homo_sapiens_assembly38.fasta file downloaded from): https://console.cloud.google.com/storage/browser/genomics-public-data/resources/broad/hg38/v0 and hg38 without alternative contigs (GCA_000001405.15_GRCh38_no_alt_analysis_set.fna.gz downloaded from): https://ftp.ncbi.nlm.nih.gov/genomes/all/GCA/000/001/405/GCA_000001405.15_GRCh38/seqs_for_alignment_pipelines.ucsc_ids/.

Declarations

Competing Interests

FM is co-inventor on patents related to cfDNA analyses. FM has consulted for Roche Dx. MK is a scientific advisor for Oncobit AG. Other authors declare no competing interests.

Funding

This study was funded by the Candoc Grant of the University of Zurich (Nr. FK-22-089) awarded to Ivna Ivankovic and the Forschungskredit Postdoc Grant of the University of Zurich awarded to Zsolt Balázs (FK-20-103). N.M. and F.M. are supported by a Dutch Cancer Fund (KWF-12822). Funders have no role in the design of the study.

Author Contributions

F.M., M.K. and Z.B. designed the study; I.I. and Z.B. performed the data analysis with contributions from T.G., N.M. and C.T. Z.B., M.K. and F.M. supervised the data analysis. I.I and Z.B. wrote the manuscript with contributions from all coauthors. I.B. organized sample collection and clinical data curation.

Acknowledgements

The authors are thankful to the Amsterdam UMC Liquid Biopsy Center for logistical support and advice. F.M. was funded by the Amsterdam UMC Liquid Biopsy Center, an initiative made possible through the Stichting Cancer Center Amsterdam. F.M. acknowledges support from the

CRUK National Biomarker Centre, the Manchester Experimental Cancer Medicine Centre, and the NIHR Manchester Biomedical Research Centre.

References

1. Dawson S-J, Tsui DWY, Murtaza M, Biggs H, Rueda OM, Chin S-F, et al.. Analysis of Circulating Tumor DNA to Monitor Metastatic Breast Cancer. *New England Journal of Medicine*. Massachusetts Medical Society; 2013; doi: 10.1056/NEJMOA1213261.
2. Bettegowda C, Sausen M, Leary RJ, Kinde I, Wang Y, Agrawal N, et al.. Detection of circulating tumor DNA in early- and late-stage human malignancies. *Sci Transl Med*. American Association for the Advancement of Science; 2014; doi: 10.1126/SCITRANSLMED.3007094.
3. Sun K, Jiang P, Chan KCA, Wong J, Cheng YKY, Liang RHS, et al.. Plasma DNA tissue mapping by genome-wide methylation sequencing for noninvasive prenatal, cancer, and transplantation assessments. *Proc Natl Acad Sci U S A*. National Academy of Sciences; 2015; doi: 10.1073/PNAS.1508736112.
4. Cristiano S, Leal A, Phallen J, Fiksel J, Adleff V, Bruhm DC, et al.. Genome-wide cell-free DNA fragmentation in patients with cancer. *Nature*. Nature Publishing Group; 2019; doi: 10.1038/s41586-019-1272-6.
5. Heitzer E, Auinger L, Speicher MR. Cell-Free DNA and Apoptosis: How Dead Cells Inform About the Living. *Trends Mol Med*. Elsevier Ltd; 2020; doi: 10.1016/j.molmed.2020.01.012.
6. Alix-Panabières C, Pantel K. Clinical applications of circulating tumor cells and circulating tumor DNA as liquid biopsy. *Cancer Discov*. American Association for Cancer Research Inc.; 2016; doi: 10.1158/2159-8290.CD-15-1483/43226/P/CLINICAL-APPLICATIONS-OF-CIRCULATING-TUMOR-CELLS.
7. Zhang YC, Zhou Q, Wu YL. The emerging roles of NGS-based liquid biopsy in non-small cell lung cancer. *J Hematol Oncol*. BioMed Central Ltd.; 2017; doi: 10.1186/S13045-017-0536-6.
8. Almodovar K, Iams WT, Meador CB, Zhao Z, York S, Horn L, et al.. Longitudinal Cell-Free DNA Analysis in Patients with Small Cell Lung Cancer Reveals Dynamic Insights into Treatment Efficacy and Disease Relapse. *Journal of Thoracic Oncology*. Elsevier; 2018; doi: 10.1016/J.JTHO.2017.09.1951.
9. Lapin M, Oltedal S, Tjensvoll K, Buhl T, Smaaland R, Garresori H, et al.. Fragment size and level of cell-free DNA provide prognostic information in patients with advanced pancreatic cancer. *J Transl Med*. BioMed Central Ltd.; 2018; doi: 10.1186/S12967-018-1677-2.
10. Mouliere F, Chandrananda D, Piskorz AM, Moore EK, Morris J, Ahlborn LB, et al.. Enhanced detection of circulating tumor DNA by fragment size analysis. *Sci Transl Med*. 2018; doi: 10.1126/scitranslmed.aat4921.
11. Sadeh R, Sharkia I, Fialkoff G, Rahat A, Gutin J, Chappleboim A, et al.. ChIP-seq of plasma cell-free nucleosomes identifies gene expression programs of the cells of origin. *Nature Biotechnology 2021 39:5*. Nature Publishing Group; 2021; doi: 10.1038/s41587-020-00775-6.
12. Snyder MW, Kircher M, Hill AJ, Daza RM, Shendure J. Cell-free DNA Comprises an In Vivo Nucleosome Footprint that Informs Its Tissues-Of-Origin. *Cell*. NIH Public Access; 2016; doi: 10.1016/j.cell.2015.11.050.

13. Shen H, Yang M, Liu J, Chen K, Li X. Development of a deep learning model for cancer diagnosis by inspecting cell-free DNA end-motifs. *npj Precision Oncology 2024 8:1*. Nature Publishing Group; 2024; doi: 10.1038/s41698-024-00635-5.
14. Jiang P, Sun K, Peng W, Cheng SH, Ni M, Yeung PC, et al.. Plasma DNA end-motif profiling as a fragmentomic marker in cancer, pregnancy, and transplantation. *Cancer Discov*. American Association for Cancer Research Inc.; 2020; doi: 10.1158/2159-8290.CD-19-0622/333461/AM/PLASMA-DNA-END-MOTIF-PROFILING-AS-A-FRAGMENTOMIC.
15. Moldovan N, van der Pol Y, van den Ende T, Boers D, Verkuijlen S, Creemers A, et al.. Multi-modal cell-free DNA genomic and fragmentomic patterns enhance cancer survival and recurrence analysis. *Cell Rep Med*. Cell Press; 2024; doi: 10.1016/J.XCRM.2023.101349.
16. van der Leest P, Boonstra PA, Elst A Ter, van Kempen LC, Tibbesma M, Koopmans J, et al.. Comparison of Circulating Cell-Free DNA Extraction Methods for Downstream Analysis in Cancer Patients. *Cancers 2020, Vol 12, Page 1222*. Multidisciplinary Digital Publishing Institute; 2020; doi: 10.3390/CANCERS12051222.
17. Van Der Pol Y, Moldovan N, Verkuijlen S, Ramaker J, Boers D, Onstenk W, et al.. The Effect of Preanalytical and Physiological Variables on Cell-Free DNA Fragmentation. *Clin Chem*. Oxford Academic; 2022; doi: 10.1093/CLINCHEM/HVAC029.
18. Shtumpf M, Piroeva K V., Agrawal SP, Jacob DR, Teif VB. NucPosDB: a database of nucleosome positioning in vivo and nucleosomics of cell-free DNA. *Chromosoma*. Chromosoma; 2022; doi: 10.1007/S00412-021-00766-9.
19. Bao R, Huang L, Andrade J, Tan W, Kibbe WA, Jiang H, et al.. Review of current methods, Applications, And data management for the bioinformatics analysis of whole exome sequencing. *Cancer Inform*. Libertas Academica Ltd.; 2014; doi: 10.4137/CIN.S13779/ASSET/IMAGES/LARGE/10.4137_CIN.S13779-FIG4.JPEG.
20. Del Fabbro C, Scalabrin S, Morgante M, Giorgi FM. An Extensive Evaluation of Read Trimming Effects on Illumina NGS Data Analysis. *PLoS One*. Public Library of Science; 2013; doi: 10.1371/JOURNAL.PONE.0085024.
21. Benjamini Y, Speed TP. Summarizing and correcting the GC content bias in high-throughput sequencing. *Nucleic Acids Res*. Oxford Academic; 2012; doi: 10.1093/NAR/GKS001.
22. Pabinger S, Dander A, Fischer M, Snajder R, Sperk M, Efremova M, et al.. A survey of tools for variant analysis of next-generation genome sequencing data. *Brief Bioinform*. Oxford Academic; 2014; doi: 10.1093/BIB/BBS086.
23. Valiente-Mullor C, Beamud B, Ansari I, Frances-Cuesta C, Garcia-Gonzalez N, Mejia L, et al.. One is not enough: On the effects of reference genome for the mapping and subsequent analyses of short-reads. *PLoS Comput Biol*. Public Library of Science; 2021; doi: 10.1371/JOURNAL.PCBI.1008678.
24. Wang H, Mennea PD, Chan YKE, Cheng Z, Neofytou MC, Surani AA, et al.. A standardized framework for robust fragmentomic feature extraction from cell-free DNA sequencing data. *Genome Biol*. BioMed Central Ltd; 2025; doi: 10.1186/S13059-025-03607-5/FIGURES/7.
25. van der Pol Y, Moldovan N, Ramaker J, Bootsma S, Lenos KJ, Vermeulen L, et al.. The landscape of cell-free mitochondrial DNA in liquid biopsy for cancer detection. *Genome Biol*. BioMed Central Ltd; 2023; doi: 10.1186/S13059-023-03074-W.
26. Köster J, Rahmann S. Snakemake—a scalable bioinformatics workflow engine. *Bioinformatics*. Oxford Academic; 2012; doi: 10.1093/BIOINFORMATICS/BTS480.

27. Adalsteinsson VA, Ha G, Freeman SS, Choudhury AD, Stover DG, Parsons HA, et al.. Scalable whole-exome sequencing of cell-free DNA reveals high concordance with metastatic tumors. *Nature Communications 2017 8:1*. Nature Publishing Group; 2017; doi: 10.1038/s41467-017-00965-y.
28. Peneder P, Stütz AM, Surdez D, Krumbholz M, Semper S, Chicard M, et al.. Multimodal analysis of cell-free DNA whole-genome sequencing for pediatric cancers with low mutational burden. *Nature Communications 2021 12:1*. Nature Publishing Group; 2021; doi: 10.1038/s41467-021-23445-w.
29. Mouliere F, Chandrananda D, Piskorz AM, Moore EK, Morris J, Ahlborn LB, et al.. Enhanced detection of circulating tumor DNA by fragment size analysis. *Sci Transl Med*. Sci Transl Med; 2018; doi: 10.1126/SCITRANSLMED.AAT4921.
30. Adalsteinsson VA, Ha G, Freeman SS, Choudhury AD, Stover DG, Parsons HA, et al.. Scalable whole-exome sequencing of cell-free DNA reveals high concordance with metastatic tumors. *Nature Communications 2017 8:1*. Nature Publishing Group; 2017; doi: 10.1038/s41467-017-00965-y.
31. Ormond C, Ryan NM, Corvin A, Heron EA. Converting single nucleotide variants between genome builds: from cautionary tale to solution. *Brief Bioinform*. Oxford Academic; 2021; doi: 10.1093/BIB/BBAB069.
32. Chen M, Zhao H. Next-generation sequencing in liquid biopsy: cancer screening and early detection. *Hum Genomics*. NLM (Medline); 2019; doi: 10.1186/S40246-019-0220-8.
33. Cameron JM, Sala A, Antoniou G, Brennan PM, Butler HJ, Conn JJA, et al.. A spectroscopic liquid biopsy for the earlier detection of multiple cancer types. *British Journal of Cancer 2023 129:10*. Nature Publishing Group; 2023; doi: 10.1038/s41416-023-02423-7.
34. Zviran A, Schulman RC, Shah M, Hill STK, Deochand S, Khamnei CC, et al.. Genome-wide cell-free DNA mutational integration enables ultra-sensitive cancer monitoring. *Nature Medicine 2020 26:7*. Nature Publishing Group; 2020; doi: 10.1038/s41591-020-0915-3.
35. Zhou Q, Kang G, Jiang P, Qiao R, Lam WKJ, Yu SCY, et al.. Epigenetic analysis of cell-free DNA by fragmentomic profiling. *Proc Natl Acad Sci U S A*. NLM (Medline); 2022; doi: 10.1073/PNAS.2209852119.
36. Moss J, Magenheim J, Neiman D, Zemmour H, Loyfer N, Korach A, et al.. Comprehensive human cell-type methylation atlas reveals origins of circulating cell-free DNA in health and disease. *Nature Communications 2018 9:1*. Nature Publishing Group; 2018; doi: 10.1038/s41467-018-07466-6.
37. Jiang H, Lei R, Ding SW, Zhu S. Skewer: A fast and accurate adapter trimmer for next-generation sequencing paired-end reads. *BMC Bioinformatics*. BioMed Central Ltd.; 2014; doi: 10.1186/1471-2105-15-182.
38. Li H, Durbin R. Fast and accurate short read alignment with Burrows–Wheeler transform. *Bioinformatics*. Oxford Academic; 2009; doi: 10.1093/BIOINFORMATICS/BTP324.
39. McKenna A, Hanna M, Banks E, Sivachenko A, Cibulskis K, Kernytsky A, et al.. The Genome Analysis Toolkit: A MapReduce framework for analyzing next-generation DNA sequencing data. *Genome Res*. Cold Spring Harbor Laboratory Press; 2010; doi: 10.1101/GR.107524.110.

40. Ewels P, Magnusson M, Lundin S, Käller M. MultiQC: summarize analysis results for multiple tools and samples in a single report. *Bioinformatics*. Oxford Academic; 2016; doi: 10.1093/BIOINFORMATICS/BTW354.
41. Jun G, Wing MK, Abecasis GR, Kang HM. An efficient and scalable analysis framework for variant extraction and refinement from population scale DNA sequence data. *Genome Res*. Cold Spring Harbor Laboratory Press; 2015; doi: 10.1101/GR.176552.114.
42. Peneder P, Bock C, Tomazou EM. LIQUORICE: detection of epigenetic signatures in liquid biopsies based on whole-genome sequencing data. *Bioinformatics Advances*. Oxford Academic; 2022; doi: 10.1093/BIOADV/VBAC017.
43. Thurman RE, Rynes E, Humbert R, Vierstra J, Maurano MT, Haugen E, et al.. The accessible chromatin landscape of the human genome. *Nature 2012 489:7414*. Nature Publishing Group; 2012; doi: 10.1038/nature11232.
44. Dunham I, Kundaje A, Aldred SF, Collins PJ, Davis CA, Doyle F, et al.. An Integrated Encyclopedia of DNA Elements in the Human Genome. *Nature*. NIH Public Access; 2012; doi: 10.1038/NATURE11247.
45. Kuhn RM, Haussler D, James Kent W. The UCSC genome browser and associated tools. *Brief Bioinform*. Oxford Academic; 2013; doi: 10.1093/BIB/BBS038.
46. Li H, Handsaker B, Wysoker A, Fennell T, Ruan J, Homer N, et al.. The Sequence Alignment/Map format and SAMtools. *Bioinformatics*. Oxford Academic; 2009; doi: 10.1093/BIOINFORMATICS/BTP352.
47. Danecek P, Bonfield JK, Liddle J, Marshall J, Ohan V, Pollard MO, et al.. Twelve years of SAMtools and BCFtools. *Gigascience*. Oxford Academic; 2021; doi: 10.1093/GIGASCIENCE/GIAB008.
48. Kurtzer GM, Sochat V, Bauer MW. Singularity: Scientific containers for mobility of compute. *PLoS One*. Public Library of Science; 2017; doi: 10.1371/JOURNAL.PONE.0177459.

Figure legends:

Figure 1. Overview of study cohort and cfDNA preprocessing workflow. We analyzed the low-pass whole-genome cfDNA sequencing data of 20 healthy individuals and 20 patients with lung cancer. Following read trimming and filtering, alignment to the chosen reference genome, and post-alignment filtering using lenient or strict options, we extracted four types of features to enable the classification of healthy and cancer samples: fragment lengths, tumor fraction scores derived from copy number aberrations, and cell type signatures.

Figure 2. Variance and characteristics of studied features across different preprocessing settings.

A) Dispersion (variance divided by the mean) of examined features among 16 studied preprocessing settings. Fragment length feature displays the lowest dispersion, whereas lung epithelial cell type signature displays the highest sample dispersion across examined preprocessing settings. B) A Principal Component Analysis (PCA) plot of 6 investigated features across 16 studied preprocessing settings. Filtering and the choice of reference genome build appear to explain the most variance in the data. Trimming has the least effect. C) Correlation plots for six examined features; displaying the Pearson's correlation of feature's value obtained by one preprocessing setting with values obtained by every other preprocessing setting. The lower diagonal represents healthy samples (in green), while the upper diagonal corresponds to lung cancer samples (in purple). The color legends represent four distinct categories of investigated bioinformatics preprocessing settings: trimming, choice of reference genome, and post-alignment filtering.

Figure 3. Extracted cfDNA features in healthy and cancer samples and their differences in distinguishing healthy from cancer samples across 16 bioinformatics preprocessing settings. A) -log10(p)-values of examined features evaluated across 16 preprocessing settings. Higher values indicate better separation between healthy individuals and patients with lung cancer. The p-values were computed using a t-test. B) The area under the ROC curve (AUC) for six investigated features across 16 different bioinformatics preprocessing settings. A higher AUC value indicates better distinction between the healthy and cancer samples. Boxes represent interquartile range (25th and 75th percentiles) with the median depicted as a black line. The whiskers extend to display the minimum and maximum values

Figure 4. Coverage-dependence of the investigated features and the effects of size selection. The area under the ROC curve (AUC) for six investigated features across 16 different bioinformatics preprocessing settings. A higher AUC value indicates better distinction between

the healthy and cancer samples. 0.1x, 1x and full refer to the coverage of the samples. Size-sel: *in silico* size selection of fragments <150 bp. Boxes represent interquartile range (25th and 75th percentiles) with the median depicted as a black line. The whiskers extend to display the minimum and maximum values.

Figure 1

Click here to access/download;Figure;Figure1.p

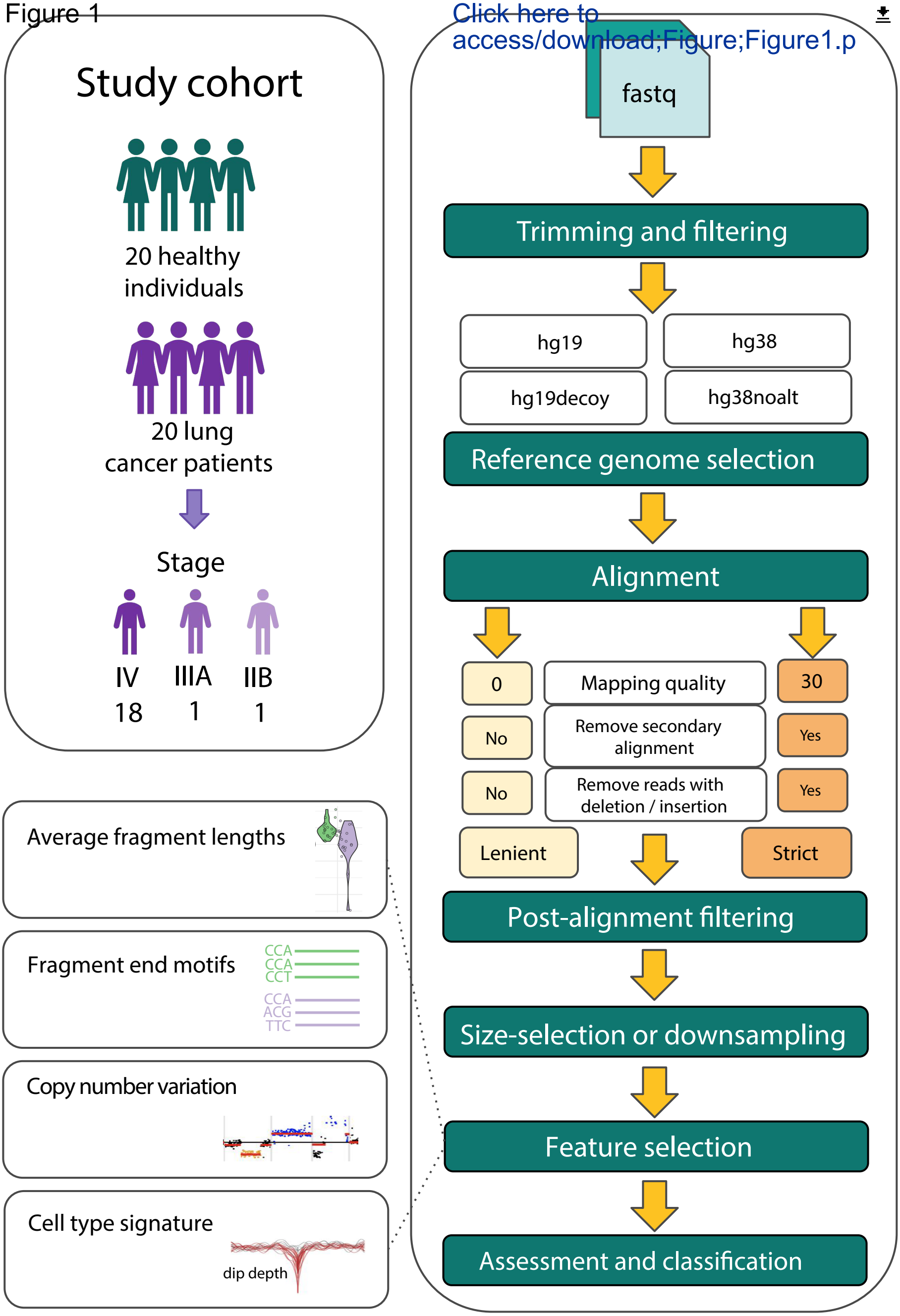

Figure 2

Click here to access/download;Figure;Figure2.pdf

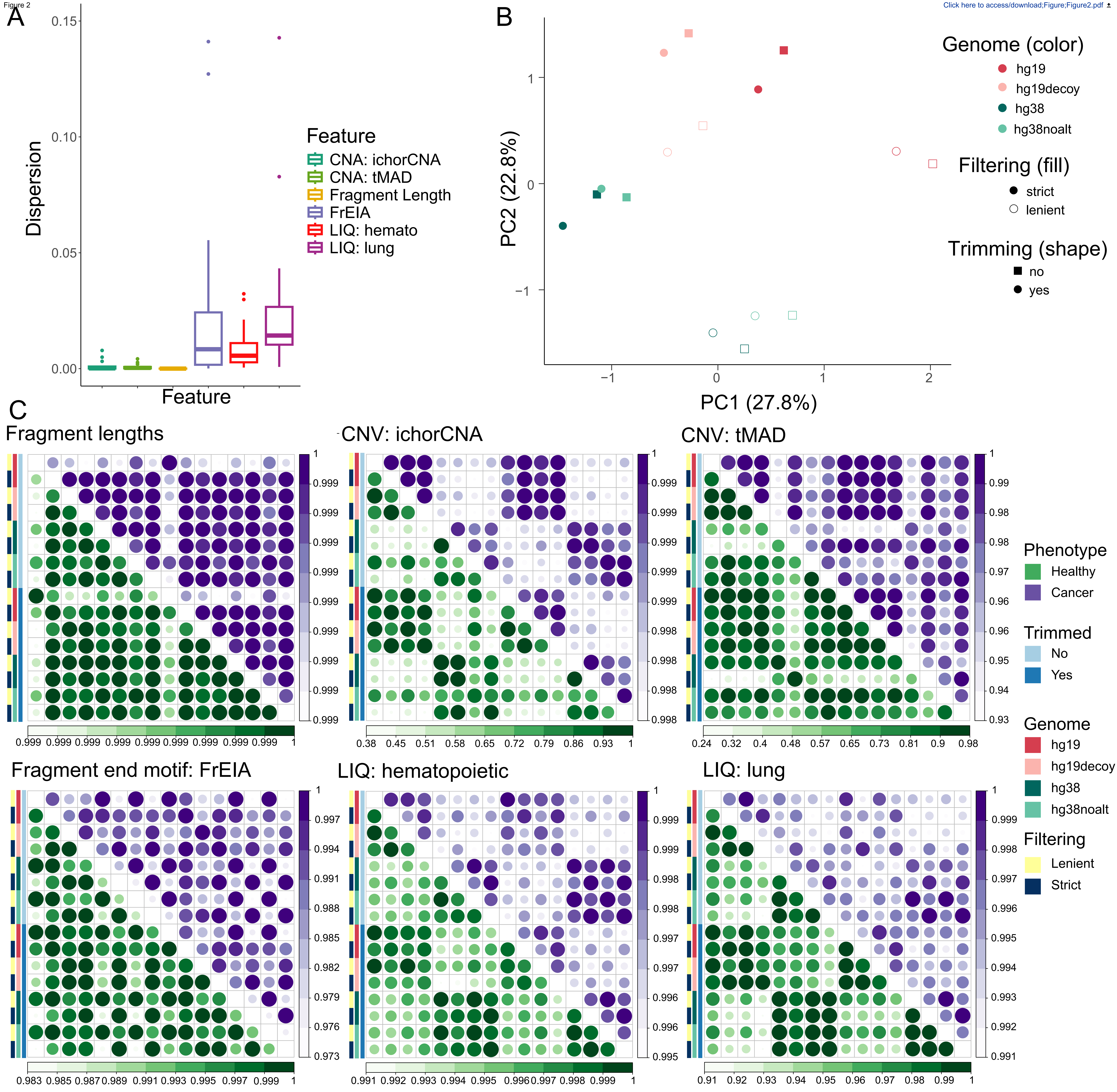

Figure 3

Click here to access/download;Figure;Figure3.pdf

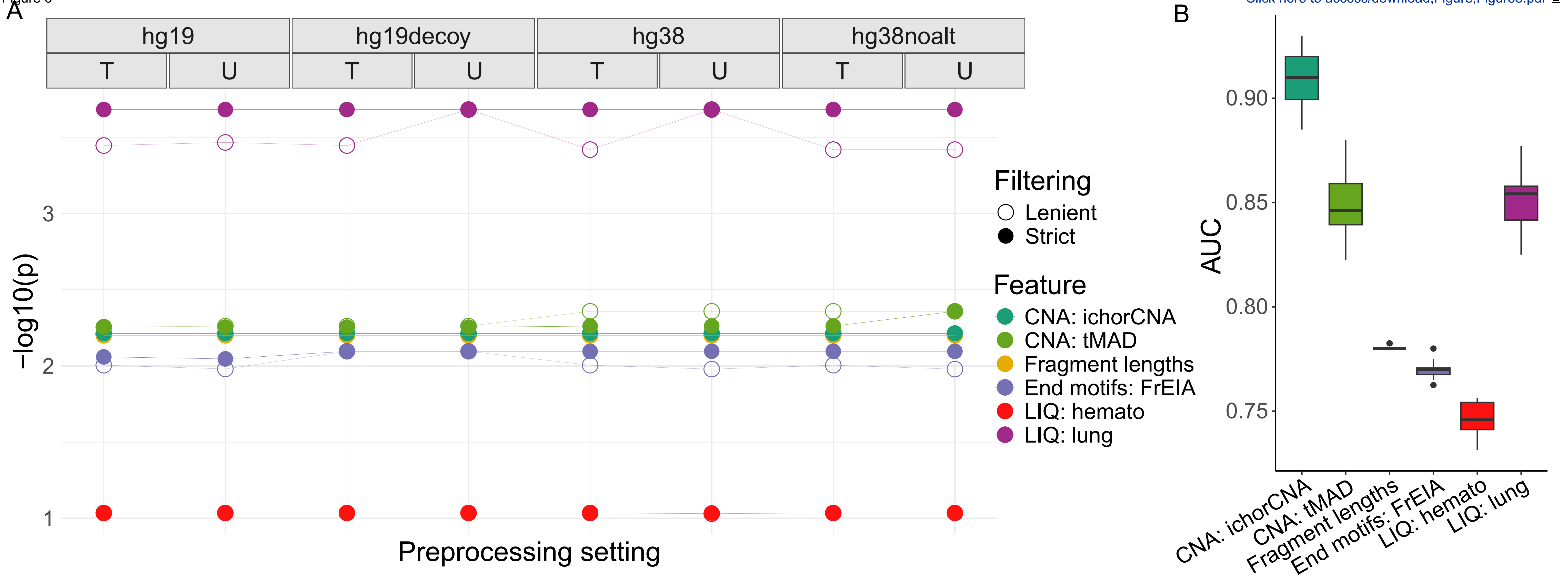

Figure 4

Click here to access/download;Figure;Figure4.pdf

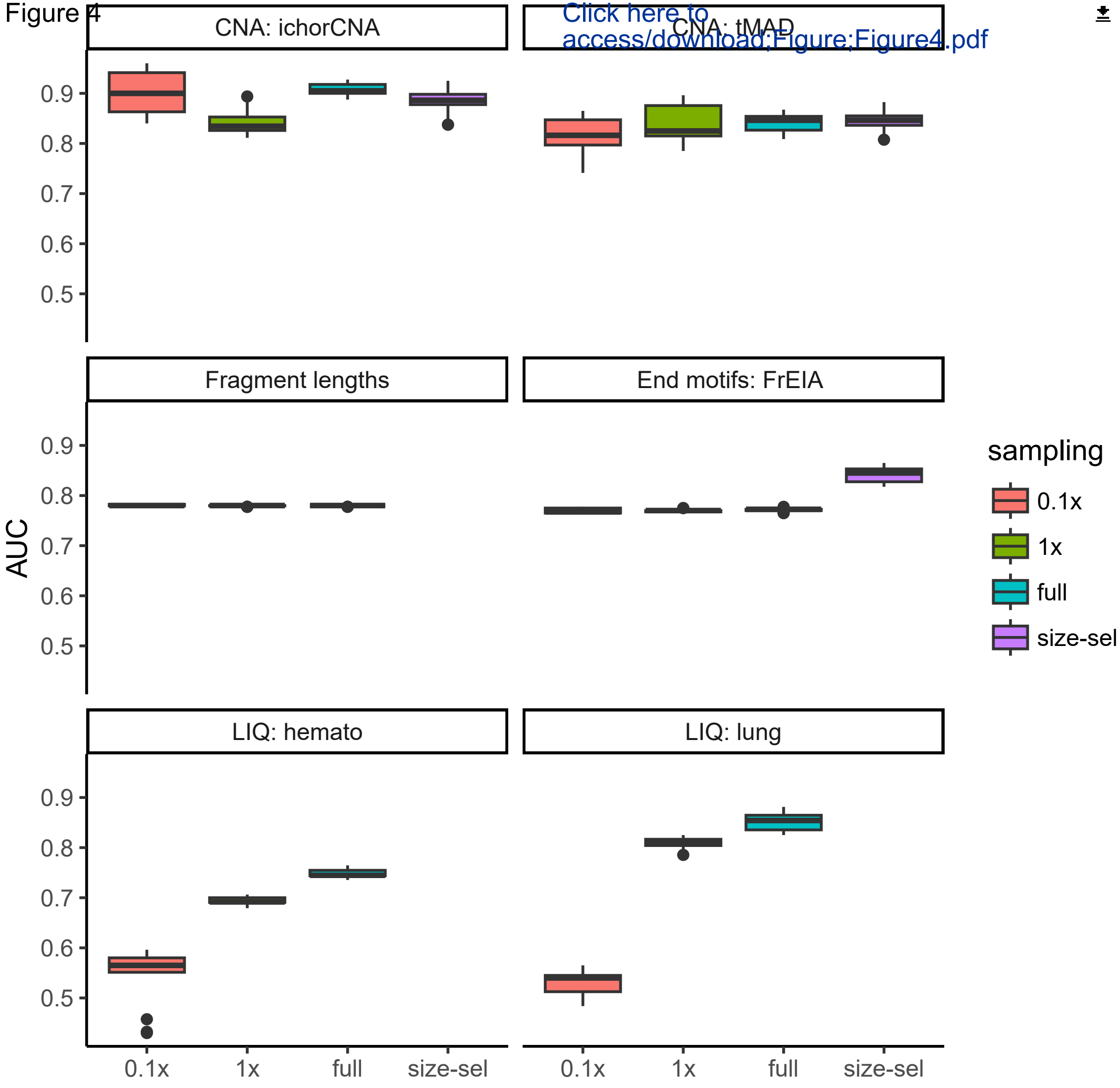

Supplementary Material

Click here to access/download
Supplementary Material
SupplementaryFigures.pdf

Personal Cover Click here to access/download;Personal Cover;coverletter.pdf

University of Zurich[UZH]

Department of Quantitative Biomedicine

University of Zurich
Winterthurerstrasse 190
CH-8057 Zurich
www.dqbm.uzh.ch

Michael Krauthammer, Prof. Dr. med.
Director
Department of Quantitative Biomedicine, University of Zurich
Chair of Medical Informatics, University Hospital Zurich
Schmelzbergstrasse 26
8006 Zurich
michael.krauthammer@uzh.ch
https://krauthammerlab.ch/

The effects of bioinformatics preprocessing on cell-free DNA fragment analysis

Zurich, 16.07.2025.

Dear Hongling Zhou, dear Editors,

We are resubmitting our manuscript entitled "The effects of bioinformatics preprocessing on cell-free DNA fragment analysis" for consideration for publication in GigaScience.

We submitted an earlier version of this manuscript to GigaScience last year. When after a round of revision, the manuscript was rejected. In response to reviewer feedback on our previous submission, we have substantially revised the manuscript to expand both the scope and depth of our analysis. You can find our detailed response to the reviewers' comments on the next page. We believe that these revisions significantly enhance the manuscript's relevance to researchers working with cell-free DNA (cfDNA) data, and that it now aligns with GigaScience's requirements.

cfDNA sequencing is increasingly used in cancer diagnostics and monitoring, yet the field lacks consensus on bioinformatics preprocessing best practices. In this study, we present cfDNA-Flow, a modular and reproducible bioinformatics pipeline, and systematically evaluate how commonly used preprocessing parameters, such as read trimming, genome build choice, post-alignment filtering, downsampling, and in silico fragment size selection, affect downstream fragmentomic and genomic analyses. Our study provides actionable recommendations for researchers working with cfDNA data and aims to guide future development of standardized preprocessing workflows. We believe that this work is an excellent fit for GigaScience, given the journal's focus on data-intensive biology and reproducibility.

We thank you for considering our work and look forward to the possibility of contributing to GigaScience.

Sincerely,
Kind regards,

Michael Krauthammer

University of Zurich[UZH]

Department of Quantitative Biomedicine

University of Zurich
Winterthurerstrasse 190
CH-8057 Zurich
www.dqbm.uzh.ch

Response to the reviewers' comments

Reviewer #1

The manuscript focuses on studying the impact of bioinformatic preprocessing on cell-free DNA fragment analysis. However, aside from suggesting the use of hg38 reference genomes, I find that there are little or no other conclusions that effectively guide the preprocessing of cfDNA sequencing data.

Analysis of preprocessing pipelines should yield conclusions that significantly impact subsequent results, such as the selection of short and long cfDNA length ranges for fragments, or the influence of sequencing depth on feature detection. However, since CNVs are highly robust features, their results are less affected by preprocessing, making this article lack a strong central theme and conclusive findings.

As I mentioned in my previous comments, the authors should either expand the scope of their analysis to include factors more susceptible to preprocessing effects or provide solid conclusions demonstrating the significant impact of preprocessing steps on specific aspects of cfDNA fragment analysis.

We expanded the range of investigated preprocessing approaches: We now include a detailed analysis of *in silico* size-selection and downsampling. Our results highlight how size selection disproportionately benefits fragment end motif–based classification of cancer samples, while downsampling adversely affects nucleosome footprinting and CNV-based analyses.

Furthermore, we also added an additional feature: fragment end motifs, and we evaluated the effects of all preprocessing settings on this feature as well.

The revised manuscript contains actionable guidance: Revising the manuscript complemented with additional analyses, we sharpened the conclusions about the effects of preprocessing on cfDNA-derived features (fragment lengths, end motifs, CNVs, nucleosome footprints). We show that combining fragment end motif analysis with *in silico* size selection, substantially improved the accuracy of classifying healthy and cancer samples, and therefore would recommend selecting for short cfDNA fragments when analyzing fragment end motifs.

There were no further comments from Reviewer #2.
