# Supplementary material for: The effects of bioinformatics preprocessing on cell-free DNA fragment analysis
Source: Gigascience. 2025 Oct 30;14:giaf139. doi: 10.1093/gigascience/giaf139 (PMC12720587; doi:10.1093/gigascience/giaf139)
Supplement: giaf139_Authors_Response_To_Reviewer_Comments_Original_Submission [file giaf139_authors_response_to_reviewer_comments_original_submission.pdf]

Author's Response To Reviewer Comments

We would like to thank you and the reviewer for the constructive feedback on our manuscript, which has helped us improve the clarity of our manuscript. Below we provide a point-by-point response to the reviewer's comments and describe the changes made in the revised version.

"1 The authors identified specific factors and metrics that affect cancer classification. For example, they noted that coverage >1x is beneficial for CNA and nucleosome footprinting, while fragment length and end motifs can be reliably used at lower sequencing depths. For the features and parameters tested in this study, I suggest that the authors provide a "recommendation table" summarizing suggested parameter settings for each feature."

We appreciate this suggestion and agree that a concise recommendation table will be highly useful for readers. We have now added Table 1 to the manuscript, which summarizes the recommended parameter settings for each tested feature. A corresponding sentence has been added to the Results section which reads:

"To provide practical guidance for future studies, we summarize our recommendations for preprocessing choices in Table 1."

"2. The current conclusion emphasizes that "the methods are feasible and most effects are limited". I believe the conclusion could be strengthened by highlighting the practical guidance for future cfDNA research. For instance, the authors could state: "We recommend that in future liquid biopsy studies, rigorous alignment filtering and annotation against the reference genome should be adopted as part of standardized preprocessing pipelines.""

We thank the reviewer for this constructive suggestion. We have revised the Discussion section to emphasize practical guidance for future cfDNA research by adding the following sentences:
"Besides emphasizing the coverage dependency of certain cfDNA features, we are also highlighting the importance of using the most complete reference genomes for CNA. Furthermore, we recommend strict alignment filtering (removal of secondary alignments and low-quality mappings) and the use of matching reference genome and annotation for regions of interest."

"3. The authors should briefly discuss the limitations of currently available cfDNA analysis pipelines (e.g., cfDNApipe and cfDNA UniFlow) and clarify how their work complements and improves upon these tools."

We included a brief discussion in the Discussion section of the revised manuscript outlining the features of cfDNApipe, cfDNA UniFlow and cfDNAPro, and describe how cfDNA-Flow differs from them. We clarify that our study complements these tools by providing systematic benchmarking of feature robustness and parameter settings, which can help guide further development. The added section reads as follows:
"We built a versatile bioinformatic pipeline, cfDNA-Flow, a reproducible workflow to analyze different cfDNA features, offering flexibility in the choice of preprocessing settings. cfDNA-flow sets itself apart from earlier pipelines such as cfDNApipe [31] and cfDNAPro [24] by being implemented in Snakemake, a reproducible workflow management system. The recent cfDNA UniFlow [32] is also implemented in Snakemake, however, it focuses more on extracting GC-bias corrected coverage values from genomic regions (while also running ichorCNA). In contrast, cfDNA-Flow enables the extraction of a wider range of cfDNA-related features using third-party applications. Overall, cfDNA-Flow is designed to facilitate benchmarking studies using whole-genome cfDNA-sequencing data."

"4. The section "Size selection" ends with a comma."

We thank the reviewer for pointing out this oversight. We corrected the punctuation and rephrased the sentence as follows:
"Most interestingly still, we found that cancer-healthy classification based on fragment end motifs was substantially improved by size selection. We attribute this result to the selective enrichment of cancer-specific fragment end motifs of shorter reads. The detection of these motifs was not hindered by the very low coverage of the dataset as fragment end motif analysis is generally robust to coverage."

We believe these changes have strengthened the manuscript by making it more practical, forward-looking, and relevant to both researchers and clinicians working with cfDNA.

Thank you again for your consideration.

Sincerely,
Michael Krauthammer

Cookie Preference Center

We use cookies which are necessary to make our site work. We may also use additional cookies to analyze, improve and personalize our content and your digital experience. For more information, see our Cookie Policy.

You may choose not to allow some types of cookies. However, blocking some types may impact your experience of our site and the services we are able to offer. See the different category headings below to find out more or change your settings.

Allow all

Manage Consent Preferences

Strictly Necessary Cookies

Always active

These cookies are necessary for the website to function and cannot be switched off in our systems. They are usually only set in response to actions made by you which amount to a request for services, such as setting your privacy preferences, logging in or filling in forms. You can set your browser to block or alert you about these cookies, but some parts of the site will not then work.

Functional Cookies

These cookies enable the website to provide enhanced functionality and personalisation. They may be set by us or by third party providers whose services we have added to our pages. If you do not allow these cookies then some or all of these services may not function properly.

Performance Cookies

These cookies allow us to count visits and traffic sources so we can measure and improve the performance of our site. They help us to know which pages are the most and least popular and see how visitors move around the site.

Targeting Cookies

These cookies may be set through our site by our advertising partners. They may be used by those companies to build a profile of your interests and show you relevant adverts on other sites. If you do not allow these cookies, you will experience less targeted advertising.

Cookie List

Clear

Apply Cancel

Consent Leg.Interest

Confirm my choices

Powered by onetrust
