# Supplementary material for: The effects of bioinformatics preprocessing on cell-free DNA fragment analysis
Source: Gigascience. 2025 Oct 30;14:giaf139. doi: 10.1093/gigascience/giaf139 (PMC12720587; doi:10.1093/gigascience/giaf139)
Supplement: giaf139_Reviewer_1_Report_Original_Submission — Wei Zhang -- 8/17/2025 [file giaf139_reviewer_1_report_original_submission.pdf]

Reviewer Report

Title: The effects of bioinformatics preprocessing on cell-free DNA fragment analysis

Version: Original Submission **Date:** 8/17/2025

Reviewer name: Wei Zhang

Reviewer Comments to Author:

Review comments for "The effects of bioinformatics preprocessing on cell-free DNA fragment analysis"
In this manuscript, the authors evaluated preprocessing steps for cfDNA sequencing data and explored how these steps influence downstream analyses. They performed a systematic comparison, provided detailed numerical results, and implemented a reproducible analysis workflow using Snakemake. This work is valuable for extracting cancer-derived features from cfDNA. Before publication, I have a few minor comments that should be addressed.

1. The authors identified specific factors and metrics that affect cancer classification. For example, they noted that coverage >1x is beneficial for CNA and nucleosome footprinting, while fragment length and end motifs can be reliably used at lower sequencing depths. For the features and parameters tested in this study, I suggest that the authors provide a "recommendation table" summarizing suggested parameter settings for each feature.
2. The current conclusion emphasizes that "the methods are feasible and most effects are limited". I believe the conclusion could be strengthened by highlighting the practical guidance for future cfDNA research. For instance, the authors could state: "We recommend that in future liquid biopsy studies, rigorous alignment filtering and annotation against the reference genome should be adopted as part of standardized preprocessing pipelines."
3. The authors should briefly discuss the limitations of currently available cfDNA analysis pipelines (e.g., cfDNApipe and cfDNA UniFlow) and clarify how their work complements and improves upon these tools.
4. The section "Size selection" ends with a comma.

Methods

Are the methods appropriate to the aims of the study, are they well described, and are necessary controls included? Choose an item.

Conclusions

Are the conclusions adequately supported by the data shown? Choose an item.

Reporting Standards

Does the manuscript adhere to the journal's guidelines on minimum standards of reporting? Choose an item.

Choose an item.

Statistics

Are you able to assess all statistics in the manuscript, including the appropriateness of statistical tests used? Choose an item.

Quality of Written English

Please indicate the quality of language in the manuscript: Choose an item.

Declaration of Competing Interests

Please complete a declaration of competing interests, considering the following questions:

- Have you in the past five years received reimbursements, fees, funding, or salary from an organisation that may in any way gain or lose financially from the publication of this manuscript, either now or in the future?
- Do you hold any stocks or shares in an organisation that may in any way gain or lose financially from the publication of this manuscript, either now or in the future?
- Do you hold or are you currently applying for any patents relating to the content of the manuscript?
- Have you received reimbursements, fees, funding, or salary from an organization that holds or has applied for patents relating to the content of the manuscript?
- Do you have any other financial competing interests?
- Do you have any non-financial competing interests in relation to this paper?

If you can answer no to all of the above, write 'I declare that I have no competing interests' below. If your reply is yes to any, please give details below.

I declare that I have no competing interests

I agree to the open peer review policy of the journal. I understand that my name will be included on my report to the authors and, if the manuscript is accepted for publication, my named report including any attachments I upload will be posted on the website along with the authors' responses. I agree for my report to be made available under an Open Access Creative Commons CC-BY license (http://creativecommons.org/licenses/by/4.0/). I understand that any comments which I do not wish to be included in my named report can be included as confidential comments to the editors, which will not be published.

Choose an item.

To further support our reviewers, we have joined with Publons, where you can gain additional credit to further highlight your hard work (see: https://publons.com/journal/530/gigascience). On publication of this paper, your review will be automatically added to Publons, you can then choose whether or not to claim your Publons credit. I understand this statement.

Yes Choose an item.
